# Integrated Inflammatory, Thrombo-Inflammatory, Redox and Soluble IFNAR2 Profiling During Hospitalization for COVID-19: An Exploratory Observational Cohort Study

**DOI:** 10.3390/jcm15176725

**Published:** 2026-08-29

**Authors:** Álvaro Martínez Mesa, Eva Cabrera César, María García-Fernandez, Pablo Zamorano-González, María Mercedes Segura Romero, Javier López García, Elisa Martín-Montañez, Óscar Fernández, Jose Luis Velasco Garrido

**Affiliations:** 1 Servicio de Neumología, Hospital Universitario Virgen de la Victoria, 29010 Málaga, Spain; evab.cabrera.sspa@juntadeandalucia.es (E.C.C.); mmercedes.segura.sspa@juntadeandalucia.es (M.M.S.R.); javier.lopez.garcia.sspa@juntadeandalucia.es (J.L.G.); josel.velasco.sspa@juntadeandalucia.es (J.L.V.G.); 2 Departamento de Farmacología y Pediatría, Facultad de Medicina, Instituto de Investigación Biomédica de Málaga y Plataforma en Nanomedicina—IBIMA Plataforma BIONAND, Universidad de Málaga, 29010 Málaga, Spain; oscar.fernandez.sspa@gmail.com; 3 Departamento de Fisiología Humana, Facultad de Medicina, Instituto de Investigación Biomédica de Málaga y Plataforma en Nanomedicina—IBIMA Plataforma BIONAND, Universidad de Málaga, 29010 Málaga, Spain; igf@uma.es (M.G.-F.); pablozglez@uma.es (P.Z.-G.); 4 Grupo de Investigación de Neumología, Instituto de Investigación Biomédica de Málaga y Plataforma en Nanomedicina—IBIMA Plataforma BIONAND, Universidad de Málaga, 29010 Málaga, Spain

**Keywords:** COVID-19, biomarkers, cytokines, ferritin, D-dimer, oxidative stress, sIFNAR2, prognosis

## Abstract

**Background**: Severe COVID-19 reflects a multi-layered host response with systemic inflammation, thrombo-inflammation, tissue damage, oxidative stress and altered antiviral interferon biology. We performed an integrated exploratory analysis of first-wave hospitalized patients to identify biomarker patterns associated with adverse clinical evolution during established admission. **Methods**: We analyzed 60 hospitalized COVID-19 patients and 18 healthy controls for soluble IFNAR2 (sIFNAR2) comparison. Biomarker samples were obtained during hospitalization, approximately seven days after symptom onset. Outcomes were final clinical status, severe respiratory involvement, post-sampling clinical worsening and death. Analyses included non-parametric testing, false-discovery-rate adjustment, effect-size estimation, exploratory ROC curves, parsimonious regression, penalized internal validation, composite scores and molecular-structure analyses. **Results**: Final status was favorable outcome in 31 patients, severe non-fatal disease in 22 and death in 7. sIFNAR2 was higher in patients than in healthy controls and highest among non-survivors, but did not distinguish favorably from severe non-fatal disease. The most consistent severity-associated signals were IL-6, D-dimer, total thiols, IL-10, LDH, ferritin, leukocytes and IL-1RA. D-dimer, IL-6 and ferritin yielded the largest exploratory univariable AUCs for severe respiratory involvement, whereas ferritin, IL-10, IL-1RA and sIFNAR2 predominated in event-limited mortality analyses, which were based on only seven deaths. Composite multi-axis scores and PLS-DA were tools requiring external validation. **Conclusions**: Biomarker patterns during admission were associated with adverse evolution. Conventional markers remained the most practical signals, while cytokines, redox markers and sIFNAR2 provided complementary biological information. sIFNAR2 should be interpreted as an exploratory complementary marker of the interferon receptor axis, mainly linked to mortality, not as a stand-alone clinical test or functional measure of IFNAR signaling.

## 1. Introduction

Coronavirus disease 2019 (COVID-19) provided a uniquely informative model of severe viral pneumonia in which clinical deterioration is driven by the interaction between viral replication, antiviral host responses, systemic inflammation, endothelial dysfunction, coagulation, tissue injury and oxidative stress. During the first pandemic wave, hospitalized patients often progressed from hypoxemic pneumonia to severe respiratory failure, multiorgan dysfunction and death. Early clinical cohorts identified age, comorbidity, lymphopenia, elevated C-reactive protein (CRP), ferritin, D-dimer and lactate dehydrogenase (LDH) as reproducible markers of adverse outcome [1,2]. Although vaccination, population immunity and viral evolution have modified the current epidemiology of SARS-CoV-2, first-wave cohorts remain highly relevant for understanding the biology of severe viral immunopathology and for designing preparedness strategies for future respiratory viral pandemics.

A large body of evidence supports the role of cytokines and innate immune mediators in COVID-19 severity. IL-6, IL-8 and TNF-alpha measured at hospital presentation were associated with disease severity and survival in large, hospitalized cohorts [3]. Longitudinal immune profiling identified divergent trajectories in severe disease and highlighted IL-1 receptor antagonist (IL-1RA) and IL-10 as markers of disease progression, counter-regulation and immune dysfunction [4,5]. Meta-analyses and systematic reviews have consistently reported higher IL-6, IL-10, D-dimer, ferritin, LDH and hematological inflammatory indices in severe or fatal COVID-19 [6,7,8,9]. Recent studies and reviews continue to support the prognostic value of these markers while emphasizing that isolated cytokine measurements are limited by sampling time, assay heterogeneity, treatment exposure and disease-stage dependence [10,11,12,13,14,15].

Oxidative stress and tissue injury represent a complementary axis of COVID-19 pathobiology. Total thiols reflect systemic antioxidant reserves and are typically depleted in inflammatory and oxidative states. In this clinical setting, total thiols, ferritin and LDH were identified as practical biomarkers of acute lung injury and COVID-19 severity in a previous analysis [16]. More recent systematic review evidence indicates that COVID-19 is associated with reduced thiol concentrations and that lower thiol levels are particularly evident in severe and critical disease [17]. These findings support the concept that severe COVID-19 combines inflammatory activation with impaired redox buffering capacity, endothelial dysfunction and tissue damage [18,19,20].

The type I interferon pathway is central to antiviral immunity against SARS-CoV-2. Severe COVID-19 has been associated with impaired early interferon responses, inborn errors of type I interferon immunity and neutralizing autoantibodies against type I interferons [21,22,23,24]. Host genetic studies have implicated IFNAR2, TYK2 and other interferon/JAK-STAT pathway components in critical illness susceptibility [25,26,27]. Soluble IFNAR2 (sIFNAR2), a circulating component of the interferon alpha/beta receptor axis, is therefore biologically plausible as a marker of interferon-pathway regulation. However, sIFNAR2 measurement should not be interpreted as equivalent to inherited receptor expression, cell-surface receptor density or functional interferon signaling; it is a circulating readout whose meaning may vary with disease stage, biological matrix, assay specificity and soluble isoform composition [28,29,30,31,32].

The present study was designed as an integrated exploratory analysis of a hospitalized first-wave COVID-19 cohort with a broad biomarker panel measured during admission. We aimed to define whether adverse clinical evolution was associated with a coordinated multi-axis profile comprising conventional admission markers, cytokines, thrombo-inflammatory markers, oxidative stress markers and sIFNAR2. We also aimed to delimit the added value and limitations of sIFNAR2 within this integrated framework—as an exploratory marker of the interferon receptor axis, not as a stand-alone diagnostic or prognostic test or as a direct readout of membrane IFNAR signaling.

## 2. Materials and Methods

### 2.1. Study Design and Participants

**Figure 1:**
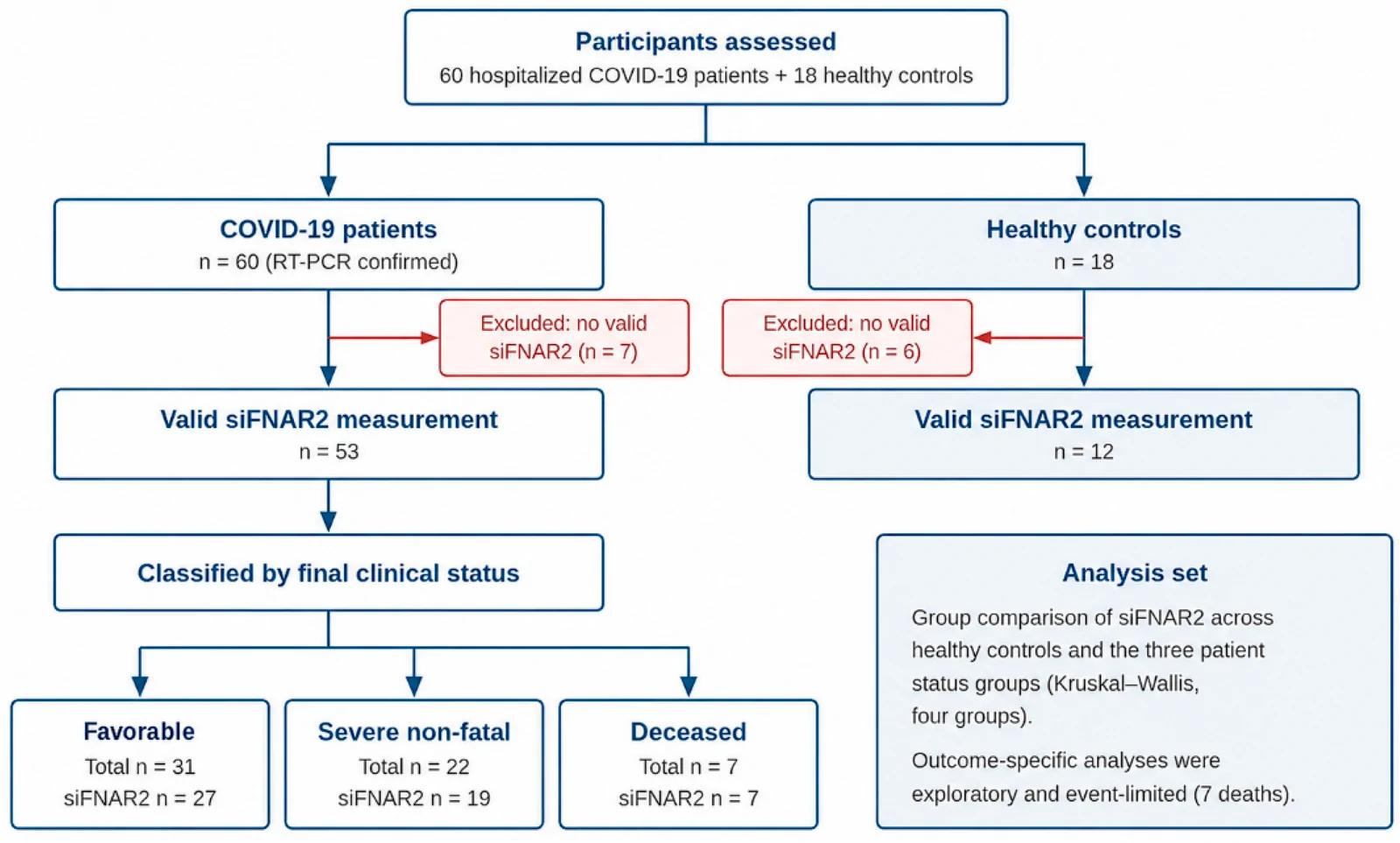
Participant flow diagram of hospitalized COVID-19 patients and healthy controls included in the sIFNAR2 analysis. The study assessed 60 RT-PCR-confirmed hospitalized COVID-19 patients and 18 healthy controls. Valid sIFNAR2 measurements were available in 53 patients and 12 controls. COVID-19 patients were classified according to final clinical status as favorable outcome, severe non-fatal disease or death. Healthy controls were included only for sIFNAR2 comparison and were not included in prognostic models. sIFNAR2, soluble interferon-α/β receptor subunit 2.

We performed an exploratory observational analysis of a single-center cohort of hospitalized patients with COVID-19 from the first pandemic wave in 2020. The analytical dataset included 60 hospitalized COVID-19 patients and 18 healthy controls recruited for sIFNAR2 comparison (Figure 1). This study represents an extension of a previously published work performed in the same cohort [16]. Sixty adult patients aged ≥18 years were eligible for inclusion if they had SARS-CoV-2 infection confirmed by rRT-PCR using the cobas^®^ test on nasopharyngeal swab, sputum, or bronchoalveolar lavage samples, together with symptoms of pneumonia or severe respiratory involvement. Patients with positive rRT-PCR results were excluded when asymptomatic or when symptoms were not accompanied by radiological findings compatible with pneumonia or severe respiratory involvement. The previous report focused on acute lung injury biomarkers, particularly total thiols, ferritin and LDH, whereas the present manuscript adds serum sIFNAR2, a broader cytokine/immune panel, final clinical status categories, post-sampling outcomes and exploratory multi-axis modeling. Healthy controls were adult volunteers of both sexes, without known prior respiratory disease, symptoms of acute infection, or other known conditions that could alter serum sIFNAR2 levels at the time of sampling. They were included solely to provide a no-infection reference range for serum sIFNAR2. Comparisons involving this group should therefore be interpreted as exploratory reference comparisons and do not support any claim of diagnostic separation. Because the biomarker sample was obtained during hospitalization rather than at emergency department presentation, values were interpreted primarily as markers of biological state during established admission and as exploratory predictors of subsequent clinical course, not as pure emergency-triage biomarkers. All study procedures were performed in accordance with the Declaration of Helsinki. The protocol was reviewed and approved by the Human Research Ethics Committee of Malaga University Hospital (approval code 01-2020; approved on 7 May 2020). Informed consent was obtained from all participants prior to enrolment. Consecutive recruitment took place between 15 March and 30 April 2020.

### 2.2. Biomarker Panel

The integrated panel included conventional admission markers, study-sample laboratory markers, cytokines, immune mediators, oxidative stress markers and sIFNAR2.

The sIFNAR2 variable was the prespecified soluble IFNAR2 ELISA measurement recorded in the analytical database and reported in ng/mL. Serum sIFNAR2 levels were determined using a commercially available colorimetric sandwich ELISA (Human IFNAR2 ELISA Kit, Abcam, Cat# ab264610, Cambridge, UK), according to the manufacturer’s instructions. Optical density was measured at 450 nm. The assay uses a pre-coated 96-well microplate format and is validated by the manufacturer for human serum, heparin plasma and cell lysate samples. The manufacturer-reported calibration range is 46.875–3000 pg/mL, with analytical sensitivity of 3.72 pg/mL. Manufacturer-reported precision for serum samples includes an intra-assay coefficient of variation of 5.0% and an inter-assay coefficient of variation of 2.3%, with reported recovery in serum of 100% (range 96–109%). Serum samples were diluted 1:8 using the sample diluent provided with the kit, and final concentrations were corrected for the corresponding dilution factor. Concentrations were calculated by interpolation from the IFNAR2 standard curve after background subtraction. Values below the assay detection/quantification limit were not extrapolated beyond the standard curve and were not included in the quantitative sIFNAR2 analysis. These samples could not be re-assayed in a more concentrated form because sufficient remaining serum volume was not available. A serum aliquot from the second blood extraction, collected in March–April 2020, was stored at −80 °C until measurement in early 2025. Before the measurement, aliquots were transferred to −20 °C for 24 h and then kept at 4 °C for 12 h before the assay was performed according to the manufacturer’s protocol. Detailed retrospective plate/batch allocation and lot-level traceability were not available in the analytical dataset and are acknowledged as analytical limitations.

The cytokine and immune biomarker panel included IL-6, IL-10, IL-1RA, IL-1beta, IL-12, IL-17, IFN-beta, TNF-alpha, TNF-beta, IFN-gamma, IL-13, IL-4. Serum concentrations were measured using a ProcartaPlex multiplex immunoassay (Thermo Fisher Scientific, Waltham, MA, USA), concentrations are reported as pg/mL. The redox/tissue-damage and endothelial/matrix-remodeling panel included total thiols, ferritin, D-dimer, LDH, leukocyte and lymphocyte counts, MMP-9 and the receptor for advanced glycation end products (RAGE) [16]. D-dimer is reported in ng/mL using DDU calibration.

### 2.3. Clinical Outcomes and Temporal Framework

The primary endpoint was final clinical status at the end of hospitalization, defined as favorable outcome, severe non-fatal disease, or death. The primary analysis compared biomarker distributions across these three categories. The binary comparison of severe/death versus favorable outcome and analyses of severe respiratory involvement, post-sampling clinical worsening, and mortality were secondary exploratory analyses. Final clinical status was assigned at the end of the hospitalization episode. Patients who survived to hospital discharge without experiencing a potentially life-threatening complication were classified as having a favorable outcome. Patients who survived to discharge after experiencing a potentially life-threatening complication during hospitalization were classified as having severe non-fatal disease. Death was defined as in-hospital death from any cause. Assignment to the severe non-fatal category was not based solely on the study-defined severe respiratory involvement endpoint; final clinical status reflected the overall clinical course and could incorporate both respiratory and non-respiratory complications. In the original study, severe respiratory involvement was defined according to the American-European Consensus Conference criteria [33]: impaired gas exchange with a PaO_2_/FiO_2_ ratio < 200, bilateral infiltrates in both lung fields on chest X-ray, and absence of evidence of left atrial hypertension. In accordance with routine clinical practice, a cardiogenic origin of the infiltrates was excluded on clinical grounds, complemented by transthoracic echocardiography whenever a cardiogenic contribution was clinically or radiologically suspected. Invasive pulmonary capillary wedge pressure measurement (<18 mmHg) was performed only in selected intensive-care patients in whom it contributed to the differential diagnosis. Throughout this manuscript, “severe respiratory involvement” refers exclusively to the study-defined physiological and radiological criteria specified above. The term “acute lung injury” is used only when referring to the previously published analysis and classification of this cohort [16]. The respiratory endpoint used in the present analysis was not formally adjudicated according to the Berlin definition of acute respiratory distress syndrome. Worsening after the study sample was considered the most temporally informative post-sampling endpoint, but some patients already had unfavorable clinical status at sampling; therefore, associations involving final severity and severe respiratory involvement include both cross-sectional disease-state information and subsequent-outcome information. The interval from symptom onset to sampling and, when available, the time from sampling to worsening were used to support temporal interpretation. Final clinical status was assigned by the attending clinical team at the end of the hospitalization episode. Serum sIFNAR2 and the cytokine panel were measured retrospectively on stored samples and were therefore unavailable to, and could not influence, the clinical assessors. Routine laboratory parameters formed part of standard clinical care and were available to the treating clinicians.

### 2.4. Statistical Analysis

Descriptive statistics: Continuous variables were summarized as median [interquartile range] and, where informative, mean ± standard deviation. Categorical variables were summarized as counts and percentages of valid observations. Missing data were not imputed for descriptive and univariable analyses. The number of valid observations is reported for each biomarker, and variables with substantial missingness were interpreted cautiously. The Supplementary Material provides an expanded availability table and should be used to judge the strength of conclusions for each analyte.

Inferential statistics: The primary analysis compared biomarker distributions across the three final clinical-status categories using Kruskal–Wallis tests, with epsilon-squared effect sizes. Binary outcome comparisons used Mann–Whitney U tests with Cliff’s delta. Categorical associations used chi-square or exact tests as appropriate and were summarized with Cramer’s V. Benjamini–Hochberg false-discovery-rate adjustment was applied within each analysis family. Selected pairwise comparisons for sIFNAR2 were adjusted using the Holm procedure. All *p*-values are two-sided and should be interpreted as exploratory. The day from symptom onset to the study biomarker sample was summarized by outcome group and was not statistically different across final clinical status categories; it was not included as an additional covariate in the exploratory regression models because of the limited sample size and to avoid overparameterization.

Discrimination and multivariable modeling: ROC curves were calculated for selected biomarkers and outcomes. Cohort-derived Youden cut-offs were used only for exploratory interpretation and should not be considered clinically validated thresholds. Continuous predictors were log(1 + x)-transformed where appropriate and standardized before modelling. Given the sample size and the limited number of deaths, multivariable logistic regression was deliberately parsimonious. Ridge-penalized logistic regression with median imputation, standardization and repeated five-fold cross-validation was used to estimate internal discrimination; these internal AUCs are expected to remain optimistic without external validation. Calibration metrics and sensitivity analyses adjusted for detailed baseline severity, comorbidity and treatment timing were not available in the current analytical report and are required for any future clinical prediction claim. Bootstrap-based variable stability and dimensionality-reduction analyses were used only as exploratory tools.

Composite scores and molecular structure. Four exploratory composite scores were constructed: an early admission score, a biological-immune score, a thrombo-damage/redox score and a combined multi-axis score. Scores were derived as risk-oriented standardized summaries of prespecified variables; right-skewed variables were log(1 + x)-transformed where appropriate, standardized, and averaged, while protective variables such as lymphocyte count and total thiols were reversed so that higher scores consistently reflected higher biological risk. Hierarchical correlation analysis used Spearman correlations after log transformation and standardization. Patient-level clustering and exploratory partial least squares discriminant analysis (PLS-DA) were performed to visualize molecular structure, not to define validated molecular subtypes or clinical prediction tools.

Although the multiplex cytokine panel included IL-1beta, IL-12, IL-17, IFN-beta, IL-13 and IL-4, these analytes were not retained for detailed presentation in the main results because they showed either low between-group variability, substantial missingness, low detectability, or no consistent association with final clinical status or post-sampling outcomes. They are therefore reported as part of the measured cytokine panel for methodological completeness but were not interpreted as clinically informative biomarkers in this exploratory cohort.

The authors declare that generative artificial intelligence was used during manuscript preparation. ChatGPT (OpenAI; GPT-5.5 Thinking) was used to assist with language editing, structural refinement, the formatting of tables and figures, and the preparation of figure legends, based on the authors’ original drafts and author-provided content. The tool was not used to generate or modify the clinical data. All AI-assisted content was critically reviewed, verified, and approved by the authors, who take full responsibility for the final manuscript and Supplementary Materials.

## 3. Results

### 3.1. Cohort Structure and Outcomes

The primary endpoint was final clinical status, and the primary analysis compared biomarker distributions across the three outcome categories; all binary and secondary-outcome analyses were exploratory. The cohort included 60 hospitalized COVID-19 patients. Final clinical status was favorable outcome in 31 patients (51.7%), severe non-fatal disease in 22 (36.7%) and death in 7 (11.7%). Median age was 64.5 years, and 40 patients (66.7%) were men. Severe respiratory involvement occurred in 29 patients, and worsening after the study biomarker sample occurred in 22. Unfavorable status at sampling, subsequent worsening and severe respiratory involvement were strongly associated with final clinical status, whereas the time from symptom onset to study sampling was comparable across outcome groups (Table 1; Supplementary Tables S1 and S2; Supplementary Figure S1). Available clinical timing and critical-care characteristics are summarized in Supplementary Table S15.

Missingness varied across analytes and time points and was mainly explained by logistical and sample-related factors. At admission, missing data were primarily due to operational constraints during the initial weeks of the pandemic, when some measurements were not systematically performed, or due to samples arriving in suboptimal condition. At the second blood draw, missingness largely reflected limited residual serum/plasma volume available for the complete biomarker panel. Importantly, missingness was not attributable to protocol-defined exclusions related to disease severity; nevertheless, variables with substantial missingness were interpreted cautiously and are summarized in Supplementary Tables S1 and S12.

### 3.2. sIFNAR2 in Controls and Hospitalized COVID-19

**Figure 2:**
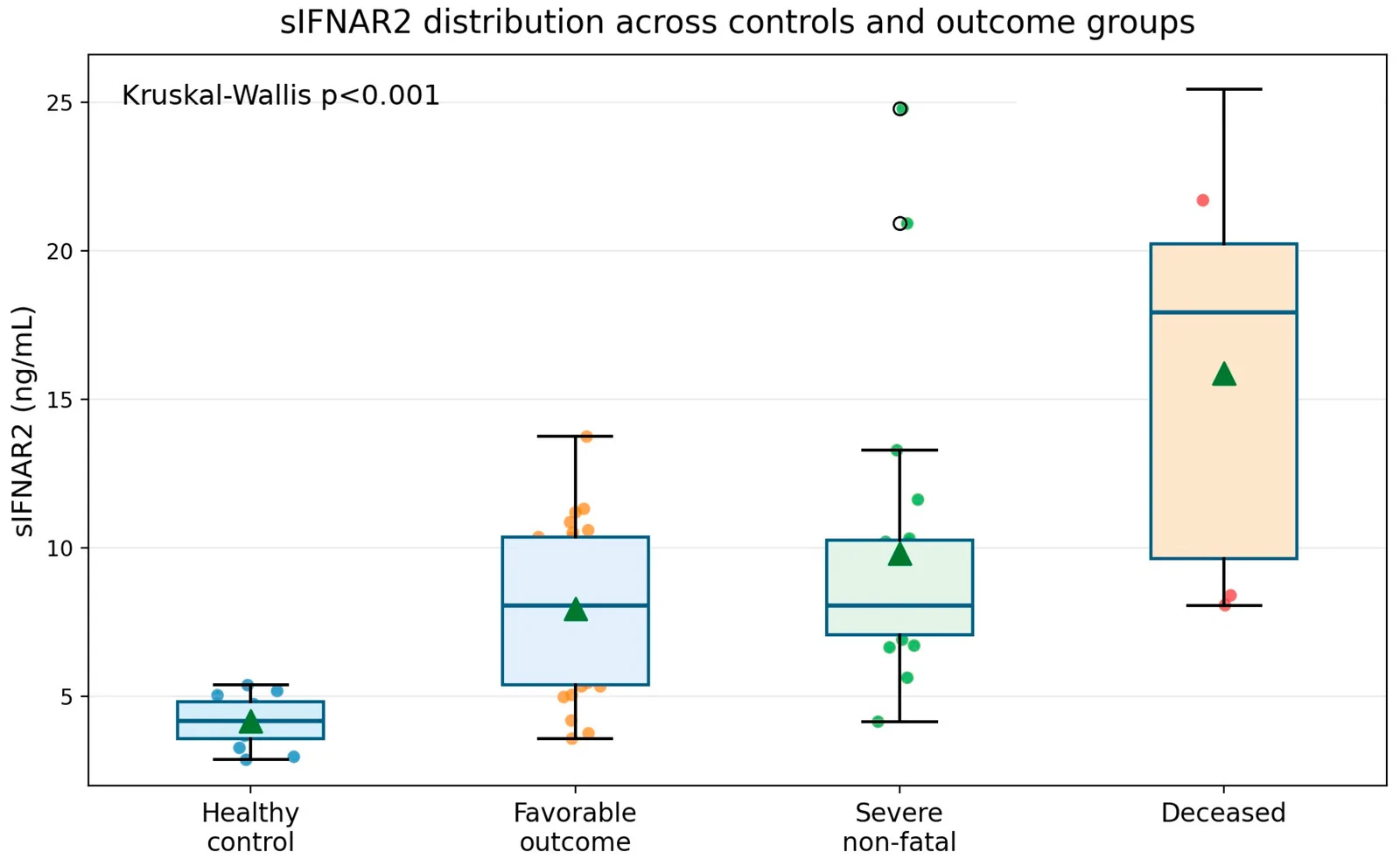
sIFNAR2 distribution across healthy controls and final clinical status groups. Boxes show interquartile ranges, horizontal lines show medians, triangles show means, whiskers show the observed range excluding plotted outliers, and points show individual observations.

Serum sIFNAR2 concentrations differed between healthy controls and hospitalized COVID-19 patients in this exploratory reference comparison and showed an upward shift among patients who died (Table 2, Figure 2, Supplementary Table S3 and Supplementary Figures S8 and S9). Eighteen controls were recruited, but valid sIFNAR2 values were available for 12 controls. Median sIFNAR2 concentrations were 4.20 ng/mL in healthy controls (*n* = 12), 8.08 ng/mL in patients with favorable outcome (*n* = 27), 8.08 ng/mL in patients with severe non-fatal disease (*n* = 19), and 17.93 ng/mL among deceased patients (*n* = 7). Pairwise analyses showed robust differences between healthy controls and each hospitalized COVID-19 group and between favorable outcome and death, whereas the difference between favorable and severe non-fatal disease was absent and the difference between severe non-fatal disease and death was borderline after multiplicity adjustment. These data support sIFNAR2 as an exploratory marker of hospitalized disease and mortality in this cohort, not as a general discriminator of non-fatal severity.

### 3.3. Biomarkers Associated with Final Clinical Status

**Figure 3:**
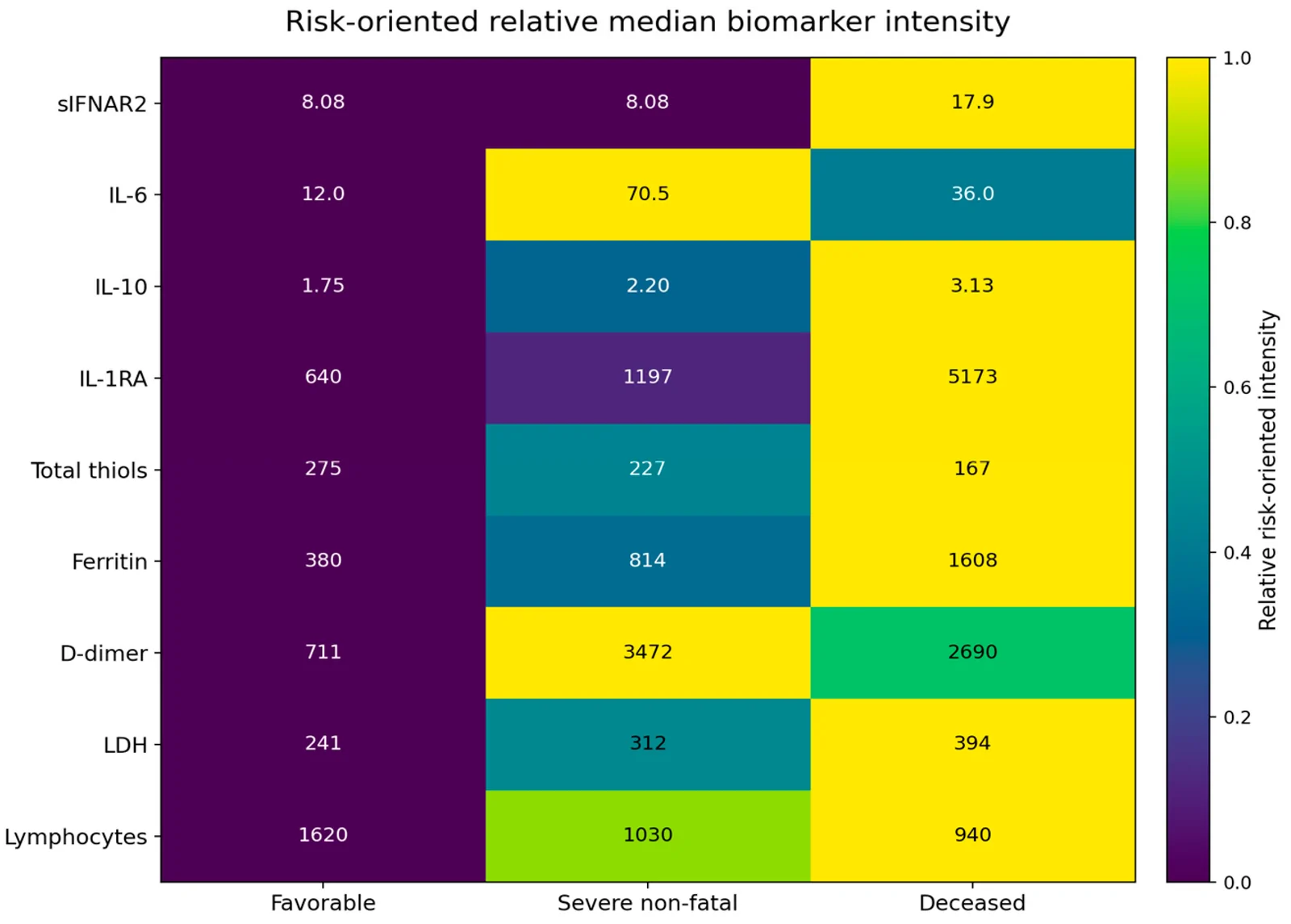
Risk-oriented relative median biomarker intensity by final clinical status. For protective markers such as total thiols and lymphocytes, lower raw values are displayed as higher risk-oriented intensity. Numbers inside cells are group medians in original units. Color scaling is marker-specific and normalized within each biomarker; color intensity therefore reflects the relative position of the outcome groups within a given marker only and does not imply comparable effect sizes or a common severity gradient across biomarkers.

The most consistent associations across final clinical status were observed for IL-6, D-dimer in the study sample, total thiols, IL-10, LDH, ferritin, leukocytes and IL-1RA (Table 2, Figure 3, Supplementary Table S4 and Supplementary Figures S2 and S10–S12). IL-6 showed the strongest global association with final status, although values were higher in severe non-fatal disease than among deceased patients, indicating that the pattern should not be interpreted as a simple linear mortality gradient. D-dimer and ferritin reflected thrombo-inflammatory and tissue-damage axes. Total thiols decreased with severity, consistent with depletion of antioxidant reserve. IL-10 and IL-1RA increased toward mortality, suggesting counter-regulatory immune activation or maladaptive immune regulation in the most adverse trajectories. sIFNAR2 retained a false-discovery-rate adjustment (FDR) significant association with final status, but its within-COVID effect size was lower than that of several inflammatory, thrombo-inflammatory and redox markers.

### 3.4. Respiratory Severity and Post-Sampling Outcomes

In binary outcome analyses, D-dimer, IL-6, ferritin, leukocytes, IL-10, IL-1RA and LDH were associated with severe respiratory involvement (Table 3; Supplementary Tables S5 and S6; Supplementary Figures S3, S4 and S8). Because this respiratory endpoint overlapped substantially with final clinical status (27 of the 29 patients with an unfavorable final status also met the respiratory endpoint), analyses based on it should be read primarily as secondary consistency analyses of the same underlying severity gradient and not as independent replication of the final-status analysis. For subsequent worsening, IL-6, IL-1RA, lower lymphocytes, ferritin and D-dimer were the most informative markers, whereas sIFNAR2 did not show robust post-sampling worsening discrimination. For mortality, ferritin, IL-10, IL-1RA and sIFNAR2 were prominent, but mortality-related AUC estimates were unstable because only seven patients died.

### 3.5. Multivariable and Composite-Score Analyses

To provide clinically interpretable effect estimates, we added a logistic regression summary for selected biomarkers (Table 4). For the severe/death composite outcome, sIFNAR2 was associated with outcome in univariable analysis but did not retain independent significance after adjustment for IL-6. For death, only univariable models were considered appropriate because only seven deaths occurred.

**Figure 4:**
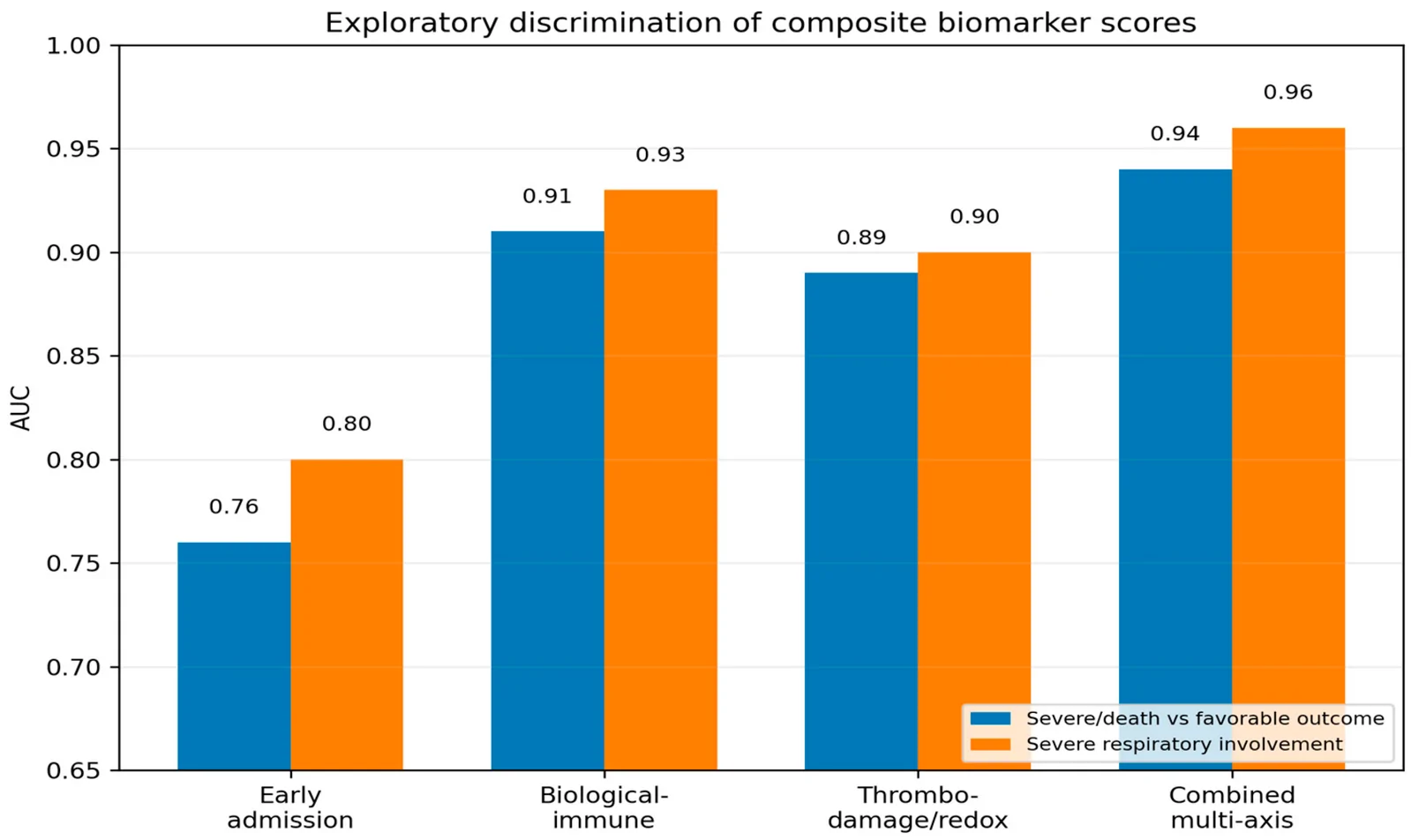
Exploratory internal discrimination of composite biomarker scores for final severity and severe respiratory involvement. AUC values are internally derived, event-limited and require external validation.

Penalized models suggested that biological and combined panels carried exploratory information about severe/death status and severe respiratory involvement (Table 5; Supplementary Tables S7 and S8; Supplementary Figures S13–S15). The early admission panel provided clinically transferable but more modest internal discrimination, while biological panels performed better for outcomes occurring after the study sample. Composite scores improved interpretability by aggregating correlated axes. However, these scores and model AUCs are internally derived, event-limited and vulnerable to optimism; they should be viewed as hypothesis-generating summaries requiring prospective external validation rather than as ready-to-use prediction tools (Figure 4).

### 3.6. Molecular Modules and Patient Clusters

**Figure 5:**
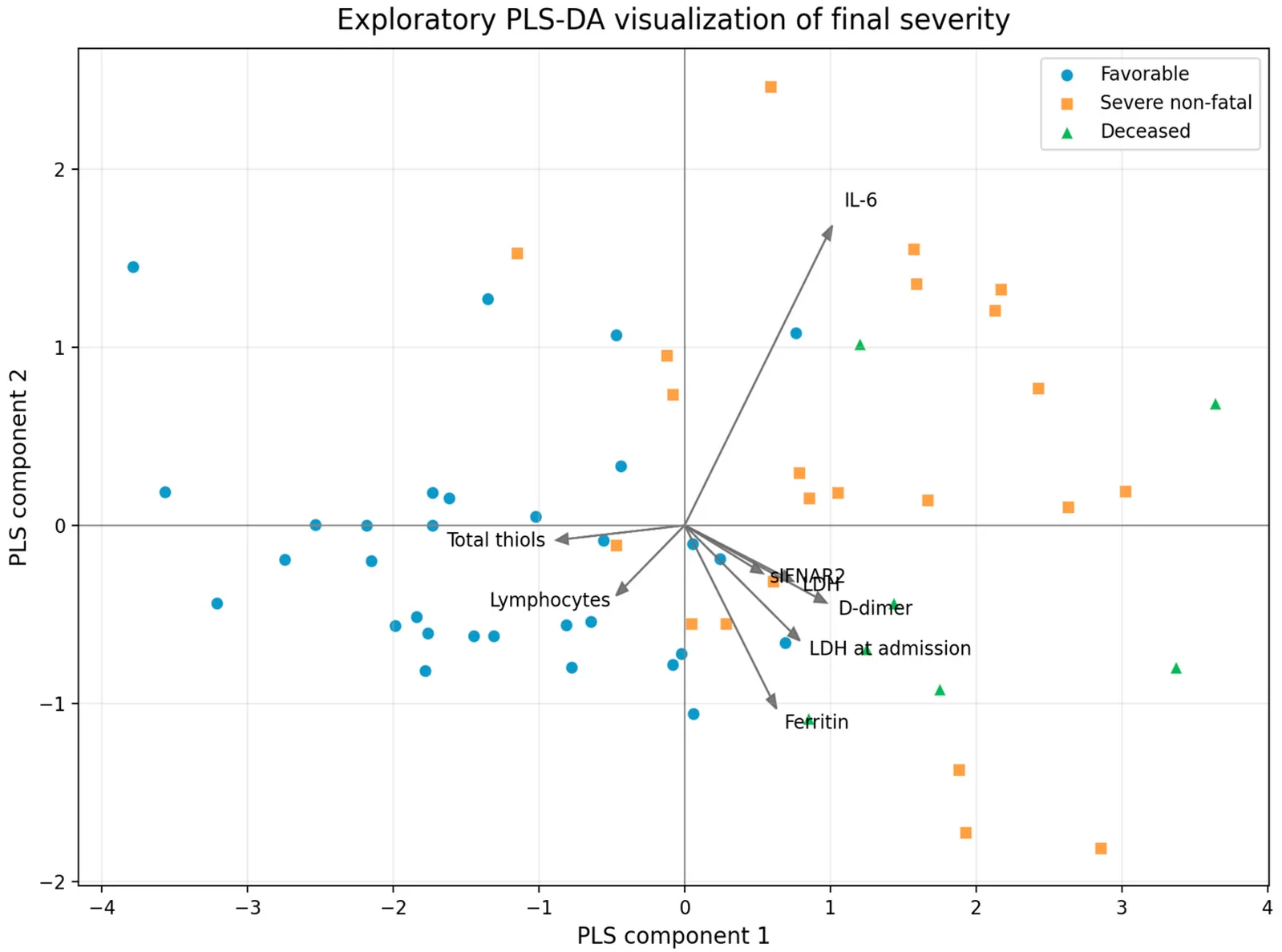
Exploratory PLS-DA projection of favorable outcome versus severe/deceased status. The analysis was used as a descriptive dimensionality-reduction tool to visualize whether the multi-axis biomarker panel carried a coherent severity-related structure. The projection should not be interpreted as a validated classifier because of the small sample size and absence of external validation.

Correlation-based analysis identified a dominant inflammation–thrombo-damage/redox module including IL-6, D-dimer, total thiols, leukocytes, ferritin, LDH, IL-10, IL-1RA, MMP-9, lymphocytes and TNF-alpha (Supplementary Table S9 and Supplementary Figures S10–S12). This module correlated with ordinal severity. sIFNAR2 formed a more independent module with a weaker but significant association with severity. Patient-level clustering separated a low-risk cluster dominated by favorable outcomes from exploratory clusters enriched for severe respiratory involvement, subsequent worsening and death. Exploratory PLS-DA provided a complementary visualization of molecular separation between favorable outcome and severe/deceased status, driven mainly by inflammatory, thrombo-damage and redox variables rather than by sIFNAR2 alone (Figure 5). Given the small sample size, these clusters and PLS-DA projections should be considered descriptive molecular patterns rather than validated clinical subtypes.

## 4. Discussion

This exploratory observational cohort supports a multi-axis interpretation of adverse evolution in hospitalized COVID-19. Rather than identifying a single dominant biomarker, the data show convergent activation of inflammatory cytokines, thrombo-inflammatory mediators, tissue-damage markers, oxidative stress biology and an exploratory interferon-receptor-axis signal. This interpretation is supported by the main figures and by the broader supplementary analyses, including variable availability, full biomarker rankings, ROC analyses, internal model performance, molecular modules, clustering and decision-curve analyses (Supplementary Tables S1–S10; Supplementary Figures S1–S15). These analyses should be interpreted as discovery-level evidence generated in a small first-wave cohort, not as a validated clinical prediction framework.

The most reproducible findings involved conventional or semi-conventional markers: IL-6, D-dimer, ferritin, LDH, leukocytes and lymphocytes. These markers have strong support in large cohorts and meta-analyses [3,6,7,8,9,11,12,13,15]. Consistent with our previous observations in this same cohort [16], the present expanded analysis places these accessible biomarkers within an integrated framework that includes additional immune markers, redox biology, sIFNAR2 and clinical-outcome-based patient classification. IL-6 was the dominant marker of final severity in our data and was associated with severe respiratory involvement and subsequent worsening, although its non-linear pattern across final-status groups emphasizes the influence of timing, treatment and biological heterogeneity. D-dimer and ferritin provided complementary thrombo-inflammatory and macrophage/tissue-damage information. LDH likely reflected lung and systemic tissue injury. Lymphopenia and leukocytosis indicated hematological immune dysregulation. These markers are not specific to COVID-19, but their accessibility and reproducibility make them pragmatic candidates for validation in future risk-stratification studies. Unlike the prior report, which focused mainly on oxidative-stress and classical inflammatory/coagulation markers, the present analysis specifically evaluates whether interferon-receptor pathway regulation, captured by sIFNAR2, provides biologically distinct information within a multi-axis framework rather than attempting to outperform established clinical biomarkers. Its incremental predictive contribution was modest, supporting a hypothesis-generating rather than a confirmatory role.

The redox axis is a distinctive strength of the analysis. Total thiols decreased with increasing severity and contributed to the dominant biomarker module. This is biologically plausible because thiols are major circulating antioxidants and their depletion reflects oxidative stress, protein oxidation and impaired buffering capacity. In a previous analysis of this same cohort, our group reported that total thiols, ferritin and LDH were associated with acute lung injury, supporting their role as biomarkers of lung damage in this clinical setting [16]. The present expanded analysis reinforces and confirms these observations that were previously reported, while extending them through the integration of additional immune biomarkers and a clinical-outcome-based classification. Moreover, recent systematic review evidence indicates that reduced thiol levels are associated with COVID-19 infection and with severe or critical disease [17]. The present data suggest that redox depletion should not be considered a secondary biochemical curiosity; it may be part of a broader host-reserve phenotype that differentiates patients who can compensate from those who progress to severe respiratory failure.

IL-10 and IL-1RA were particularly prominent in mortality analyses. This pattern should not be interpreted as simple protective anti-inflammatory activity. In severe COVID-19, elevated IL-10 and IL-1RA may reflect compensatory counter-regulation, macrophage activation, immune exhaustion or immunoparalysis. Longitudinal profiling studies have shown that IL-1RA and IL-10 are elevated early in severe disease and may help predict outcome trajectories [5]. In our cohort, their association with death suggests that mortality may be characterized by simultaneous inflammatory activation and counter-regulatory overactivation—a pattern consistent with maladaptive immune regulation rather than resolution.

sIFNAR2 was higher in hospitalized COVID-19 than in healthy controls and highest among patients who died. Nevertheless, it did not separate favorable outcome from severe non-fatal disease, was not the strongest discriminator of severe respiratory involvement, and did not robustly predict subsequent worsening. This justifies a conservative interpretation: sIFNAR2 is best positioned as an exploratory mechanistic marker of the interferon receptor axis, complementary to inflammatory and redox biomarkers, rather than as a stand-alone clinical prediction tool. The mortality signal is biologically interesting but event-limited because it is based on only seven deaths. Circulating soluble receptor concentrations may reflect soluble isoform abundance, receptor shedding, decoy activity, preserved or exhausted IFNAR-axis reserve and assay-specific epitope recognition [28,29,30,31,32].

Our sIFNAR2 results are compatible with the broader interferon literature. Severe COVID-19 has been linked to impaired type I interferon responses, inborn errors of interferon immunity and anti-interferon autoantibodies [21,22,23,24]. Host genetic studies implicate IFNAR2 and related JAK/STAT pathway components in critical illness susceptibility [25,26,27]. Published soluble IFNAR2 studies do not all address the same biological question. Yaugel-Novoa et al. [28] reported higher soluble IFNAR2 concentrations across a broad severity spectrum that included controls and severe disease, whereas Fricke-Galindo et al. [29] studied a cohort restricted to severe hospitalized disease and observed a different survival-related pattern. These apparently divergent findings are best interpreted as context-dependent rather than contradictory, because disease spectrum, sampling time, biological matrix, assay specificity and residual interferon-axis reserve may change the meaning of circulating soluble receptor concentrations. However, circulating sIFNAR2 is not a direct surrogate for genetic expression or functional antiviral signaling. Future work should therefore combine sIFNAR2 with IFNAR1/IFNAR2 genotyping, anti-type I interferon autoantibodies, interferon-stimulated gene signatures and functional stimulation assays.

The modeling results should be interpreted with caution. Composite scores and ridge-penalized models performed well internally, particularly for severe/death status and severe respiratory involvement. However, the sample size was small, events were limited, and the same cohort was used for model development and internal validation. This issue is central to the COVID-19 prognostic modelling literature: systematic reviews have shown that many published prediction models are at high risk of bias because of insufficient event counts, poor handling of missing data, heterogeneous outcome definitions and a lack of external validation [34,35]. Our results should therefore be viewed as a biologically coherent discovery analysis and as a basis for designing prospective validation studies, not as a ready-to-use clinical prediction tool.

A practical implication is that future studies should not attempt indiscriminate measurement of very large panels without a validation strategy. A rational reduced panel could combine admission LDH, CRP, ferritin, D-dimer and lymphocytes for clinically accessible risk assessment with IL-6, IL-10, IL-1RA, total thiols and sIFNAR2 for biological stratification during hospitalization. The timing of sampling is critical. In this cohort, cytokines and sIFNAR2 were measured during admission at a median of approximately seven days from symptom onset; therefore, they cannot be claimed to be emergency department triage biomarkers without prospective sampling at presentation. Serial sampling at admission, days 3–5, days 7–10, clinical worsening and recovery would be needed to distinguish baseline risk markers from dynamic progression markers.

The study has important limitations. It is single-center and exploratory, with 60 patients and only seven deaths; mortality analyses are therefore event-limited and exploratory/hypothesis-generating. The first-wave, vaccine-naive context limits direct generalizability to vaccinated populations, later SARS-CoV-2 variants and contemporary treatment pathways, although it remains informative for understanding the host response to severe natural infection. Biomarker sampling was performed during established hospitalization, not at emergency department presentation; consequently, the findings should be interpreted as disease-state and post-sampling outcome associations rather than as validated early triage tools. Treatment exposure was not randomized and is confounded by indication, especially for tocilizumab and interferon therapy. These interventions may have modified circulating cytokine concentrations and potentially influenced sIFNAR2-associated patterns. Pre-sampling treatment exposure is reported in Table 1, but exact treatment-to-sampling intervals were not sufficiently complete for robust adjustment, and statistical adjustment could not fully resolve severity-driven treatment confounding. Some patients already had unfavorable clinical status at sampling, which limits causal or purely prospective interpretation of post-sampling outcomes. The exact onset of the study-defined severe respiratory involvement endpoint was not systematically recorded, limiting precise temporal sequencing between biomarker sampling and respiratory deterioration. Critical-care and ventilatory-support data were retained as previously reported for this cohort rather than retrospectively reclassified from the current analytical dataset. Final clinical status and severe respiratory involvement were highly overlapping clinical constructs in this cohort; therefore, concordant biomarker associations across these endpoints should not be interpreted as independent confirmation, and the respiratory endpoint should be regarded as a secondary consistency analysis of the underlying severity gradient. Disease severity endpoints were based on objective physiological and radiological criteria rather than on a composite clinical score; therefore, incremental value beyond established clinical risk scores could not be formally quantified. Accordingly, the integrated biomarker panel should be interpreted as an exploratory biological stratification framework rather than as a validated clinical decision-making tool. Healthy controls were available only for sIFNAR2 comparisons. Some analytes had substantial missingness, which varied across analytes and time points and was mainly explained by logistical and sample-related factors, including early-pandemic operational constraints, suboptimal sample condition, and limited residual serum/plasma volume for the full panel at the second blood draw; in addition, several advanced models are likely optimistic despite penalization. Finally, sIFNAR2 assay standardization remains essential; lot-level traceability and plate/batch allocation were not available in the analytical dataset and should be captured prospectively in future validation studies.

The study also has strengths. It integrates conventional admission markers with cytokines, redox biology, tissue injury and interferon-axis measurement. It distinguishes admission markers from study-sample biomarkers, avoiding overstatement of emergency triage utility. It uses non-parametric statistics, effect sizes, multiplicity control, ROC analysis, penalized modeling, composite scores and molecular clustering while maintaining explicit caution about overfitting. Most importantly, it supports a biologically plausible multi-axis framework that can be tested prospectively in larger, multicenter cohorts.

## 5. Conclusions

In this first-wave hospitalized cohort, biomarker patterns measured during established admission were associated with adverse clinical evolution. The most robust signals involved inflammatory, thrombo-inflammatory, tissue-damage and redox markers, particularly IL-6, D-dimer, ferritin, LDH, IL-10, IL-1RA and total thiols. sIFNAR2 added a context-dependent exploratory signal, mainly related to mortality rather than to non-fatal severity separation; however, this finding was based on only seven deaths and should therefore be regarded as hypothesis-generating, not as a validated prognostic marker or a direct measure of IFNAR signaling. The findings are hypothesis-generating and require prospective, longitudinal and multicenter validation with standardized sampling, detailed treatment timing, calibrated prediction methods and external validation before any clinical implementation.

## Figures and Tables

**Table 1:** Cohort structure, clinical outcomes and pre-sampling treatment variables by final clinical status.

Variable	Favorable Outcome (*n* = 31)	Severe Non-Fatal (*n* = 22)	Deceased (*n* = 7)	*p* Value	Effect Size
Age, years	64.0 [56.5–72.5]	64.0 [61.2–71.8]	71.0 [59.0–75.0]	0.681	epsilon^2^ = 0.00
Days from symptom onset to study sample	5.00 [3.00–9.50]	7.00 [4.50–8.00]	7.00 [6.00–8.50]	0.766	epsilon^2^ = 0.00
Male sex	17/31 (54.8%)	18/22 (81.8%)	5/7 (71.4%)	0.117	V = 0.27
Unfavorable status at sampling	1/31 (3.2%)	10/22 (45.5%)	3/7 (42.9%)	0.000704	V = 0.49
Worsening after study sample	1/31 (3.2%)	14/22 (63.6%)	7/7 (100.0%)	4.33 × 10^−8^	V = 0.75
Severe respiratory involvement	2/31 (6.5%)	20/22 (90.9%)	7/7 (100.0%)	1.51 × 10^−10^	V = 0.87
Death	0/31 (0.0%)	0/22 (0.0%)	7/7 (100.0%)	9.36 × 10^−14^	V = 1.00
Corticosteroids before sample	9/31 (29.0%)	5/22 (22.7%)	1/7 (14.3%)	0.685	V = 0.11
Tocilizumab before sample	2/26 (7.7%)	7/18 (38.9%)	3/4 (75.0%)	0.003	V = 0.49
Interferon before sample	2/26 (7.7%)	7/18 (38.9%)	3/4 (75.0%)	0.003	V = 0.49

Continuous variables are shown as median [interquartile range, IQR]; categorical variables as *n* (%). Effect sizes: ε^2^ (epsilon-squared) for Kruskal–Wallis; V (Cramér’s V) for categorical associations. *p* values from Kruskal–Wallis or chi-square/Fisher tests as appropriate.

**Table 2:** Key biomarkers by final clinical status.

Biomarker	Unit	N	Favorable Outcome Median [IQR]	Severe Non-Fatal Median [IQR]	Deceased Median [IQR]	*p* Value	FDR q	Effect Size	Ordinal rho
IL-6	pg/mL	56	12.0 [10.0–17.0]	70.5 [31.6–164]	36.0 [29.0–87.0]	1.6 × 10^−6^	2.71 × 10^−5^	epsilon^2^ = 0.47	0.67
D-dimer, study sample	ng/mL	52	711 [358–1312]	3472 [1484–4072]	2690 [1904–3667]	1.84 × 10^−5^	0.000156	epsilon^2^ = 0.40	0.62
Total thiols	µM	55	275 [246–342]	227 [201–243]	167 [127–210]	4.19 × 10^−5^	0.000237	epsilon^2^ = 0.35	−0.61
IL-10	pg/mL	58	1.75 [1.54–2.31]	2.20 [1.97–2.95]	3.13 [2.85–3.65]	0.000356	0.002	epsilon^2^ = 0.25	0.51
LDH at admission	U/L	53	268 [235–310]	442 [314–642]	328 [310–396]	0.000400	0.002	epsilon^2^ = 0.27	0.48
Ferritin, study sample	ng/mL	40	380 [224–581]	814 [671–1437]	1608 [1313–3224]	0.000418	0.002	epsilon^2^ = 0.37	0.63
Leukocytes, study sample	cells/µL	53	6600 [4890–8420]	11,110 [7315–13,330]	10,930 [9270–12,630]	0.001	0.003	epsilon^2^ = 0.23	0.51
LDH, study sample	U/L	57	241 [212–288]	312 [258–374]	394 [298–452]	0.001	0.003	epsilon^2^ = 0.21	0.49
IL-1RA	pg/mL	48	640 [224–1699]	1197 [1009–2168]	5173 [2635–7547]	0.003	0.008	epsilon^2^ = 0.22	0.48
RAGE	ng/L	45	386 [247–741]	179 [74.0–300]	154 [34.8–321]	0.012	0.026	epsilon^2^ = 0.16	−0.44
MMP-9	µg/L	56	168 [69.5–228]	230 [213–267]	159 [150–201]	0.017	0.031	epsilon^2^ = 0.12	0.26
sIFNAR2	ng/mL	53	8.08 [5.41–10.4]	8.08 [7.08–10.3]	17.9 [9.64–20.2]	0.017	0.031	epsilon^2^ = 0.12	0.33
Leukocytes at admission	cells/µL	54	6160 [4758–9170]	6660 [4410–8310]	5680 [4460–6600]	0.820	0.820	epsilon^2^ = 0.00	−0.03

Values are median [interquartile range, IQR]. N, number of patients with available data. *p*, Kruskal–Wallis test; FDR q, Benjamini–Hochberg adjusted value; ε^2^, epsilon-squared effect size; ordinal ρ, Spearman rank correlation with ordinal clinical status. IL-6, interleukin-6; LDH, lactate dehydrogenase; sIFNAR2, soluble interferon-α/β receptor subunit 2.

**Table 3:** Biomarkers associated with secondary clinical outcomes.

Outcome	Biomarker	Unit	No-Event Median	Event Median	Cliff Delta	*p* Value	FDR q	AUC (95% CI)
Severe respiratory involvement	IL-6	pg/mL	12.0	58.0	0.77	9.64 × 10^−7^	7.76 × 10^−6^	0.88 (0.80–0.95)
Severe respiratory involvement	D-dimer, study sample	ng/mL	711	3190	0.78	1.29 × 10^−6^	7.76 × 10^−6^	0.89 (0.82–0.96)
Severe respiratory involvement	Leukocytes, study sample	cells/µL	6355	10,950	0.65	5.04 × 10^−5^	0.000101	0.83 (0.72–0.92)
Severe respiratory involvement	Ferritin, study sample	ng/mL	371	1046	0.73	9.14 × 10^−5^	0.000157	0.87 (0.74–0.97)
Severe respiratory involvement	IL-10	pg/mL	1.81	2.49	0.50	0.001	0.003	0.75 (0.59–0.85)
Severe respiratory involvement	IL-1RA	pg/mL	608	1732	0.54	0.001	0.003	0.77 (0.63–0.89)
Severe respiratory involvement	LDH, study sample	U/L	245	313	0.46	0.003	0.006	0.73 (0.61–0.86)
Severe respiratory involvement	sIFNAR2	ng/mL	7.47	9.62	0.38	0.018	0.028	0.69 (0.56–0.81)
Subsequent worsening	IL-6	pg/mL	13.0	38.0	0.55	0.000884	0.012	0.77 (0.63–0.89)
Subsequent worsening	IL-1RA	pg/mL	748	2075	0.50	0.003	0.027	0.75 (0.64–0.87)
Subsequent worsening	Lymphocytes, study sample	cells/µL	1580	965	−0.44	0.008	0.046	0.72 (0.56–0.84)
Subsequent worsening	Ferritin, study sample	ng/mL	563	1090	0.41	0.030	0.103	0.71 (0.56–0.86)
Subsequent worsening	D-dimer, study sample	ng/mL	1065	2396	0.35	0.038	0.103	0.67 (0.51–0.77)
Subsequent worsening	IL-10	pg/mL	1.94	2.40	0.32	0.048	0.114	0.66 (0.53–0.80)
Subsequent worsening	sIFNAR2	ng/mL	8.26	9.06	0.28	0.101	0.170	0.64 (0.51–0.78)
Death	IL-10	pg/mL	1.97	3.13	0.77	0.002	0.019	0.88 (0.77–0.97)
Death	Ferritin, study sample	ng/mL	646	1608	0.79	0.002	0.019	0.90 (0.79–1.00)
Death	IL-1RA	pg/mL	1115	5173	0.70	0.003	0.020	0.85 (0.70–0.98)
Death	sIFNAR2	ng/mL	8.08	17.9	0.65	0.007	0.031	0.82 (0.65–0.97)
Death	LDH, study sample	U/L	260	394	0.53	0.026	0.089	0.76 (0.54–0.92)

No-event and event medians are shown per outcome. Cliff’s delta, non-parametric effect size; *p*, Mann–Whitney U test; FDR q, Benjamini–Hochberg adjusted value; AUC, area under the ROC curve (95% confidence interval). Analyses are exploratory and event-limited.

**Table 4:** Logistic regression summary with odds ratios for selected biomarkers.

Outcome	Marker	Model	N/Events	OR per SD	95% CI	*p* Value
Severe/death vs. favorable outcome	IL-6	Univariable	56/29	9.54	2.97–30.68	0.000154
Severe/death vs. favorable outcome	D-dimer, study sample	Univariable	52/29	6.95	2.56–18.89	0.000145
Severe/death vs. favorable outcome	Ferritin, study sample	Univariable	40/29	4.49	1.50–13.47	0.007
Severe/death vs. favorable outcome	LDH, study sample	Univariable	57/29	3.38	1.61–7.10	0.0013
Severe/death vs. favorable outcome	sIFNAR2	Univariable	53/29	2.24	1.14–4.38	0.019
Severe/death vs. favorable outcome	IL-6	sIFNAR2 + IL-6	49/22	9.86	2.58–37.69	0.000821
Severe/death vs. favorable outcome	sIFNAR2	sIFNAR2 + IL-6	49/22	1.89	0.77–4.67	0.166
Death	sIFNAR2	Univariable only	53/7	4.01	1.54–10.48	0.005
Death	LDH, study sample	Univariable only	57/7	3.05	1.20–7.74	0.019
Death	Ferritin, study sample	Univariable only	40/7	16.63	1.58–174.82	0.019
Death	D-dimer, study sample	Univariable only	52/7	2.43	0.77–7.69	0.132
Death	IL-6	Univariable only	56/7	1.62	0.70–3.77	0.260

ORs are per 1-standard-deviation increase in log(1 + x)-transformed marker values. The multivariable model was restricted to sIFNAR2 plus IL-6 (*n* = 49 complete cases; 22 events; low correlation between log-transformed sIFNAR2 and IL-6, r = 0.21). No multivariable model was fitted for death because only seven events occurred. CI, confidence interval; OR, odds ratio.

**Table 5:** Summary of advanced exploratory models and composite scores.

Analysis	Outcome	Panel/Score	N	Events	Performance	Interpretation
Ridge logistic regression	Severe/death vs. favorable outcome	Admission panel	60	29	AUC 0.74 (0.69–0.77)	Internal cross-validation; exploratory
Ridge logistic regression	Severe/death vs. favorable outcome	Biological panel	60	29	AUC 0.89 (0.88–0.91)	Internal cross-validation; exploratory
Ridge logistic regression	Severe/death vs. favorable outcome	Combined panel	60	29	AUC 0.89 (0.84–0.92)	Internal cross-validation; exploratory
Ridge logistic regression	Severe respiratory involvement	Admission panel	60	29	AUC 0.86 (0.82–0.88)	Internal cross-validation; exploratory
Ridge logistic regression	Severe respiratory involvement	Biological panel	60	29	AUC 0.94 (0.91–0.95)	Internal cross-validation; exploratory
Ridge logistic regression	Severe respiratory involvement	Combined panel	60	29	AUC 0.97 (0.97–0.98)	Internal cross-validation; exploratory
Ridge logistic regression	Subsequent worsening	Admission panel	60	22	AUC 0.51 (0.45–0.56)	Internal cross-validation; exploratory
Ridge logistic regression	Subsequent worsening	Biological panel	60	22	AUC 0.69 (0.62–0.74)	Internal cross-validation; exploratory
Ridge logistic regression	Subsequent worsening	Combined panel	60	22	AUC 0.70 (0.63–0.76)	Internal cross-validation; exploratory
Ridge logistic regression	Death	Admission panel	60	7	AUC 0.76 (0.60–0.91)	Internal cross-validation; exploratory
Ridge logistic regression	Death	Biological panel	60	7	AUC 0.89 (0.85–0.92)	Internal cross-validation; exploratory
Composite score	Severe/death vs. favorable outcome	Early admission score	60	29	AUC 0.76; OR/SD 3.00	Score-level association; requires external validation
Composite score	Severe/death vs. favorable outcome	Biological-immune score	60	29	AUC 0.91; OR/SD 15.10	Score-level association; requires external validation
Composite score	Severe/death vs. favorable outcome	Thrombo-damage/redox score	60	29	AUC 0.89; OR/SD 12.20	Score-level association; requires external validation
Composite score	Severe/death vs. favorable outcome	Combined multi-axis score	60	29	AUC 0.94; OR/SD 26.22	Score-level association; requires external validation
Composite score	Severe respiratory involvement	Early admission score	60	29	AUC 0.80; OR/SD 4.10	Score-level association; requires external validation
Composite score	Severe respiratory involvement	Biological-immune score	60	29	AUC 0.93; OR/SD 12.90	Score-level association; requires external validation
Composite score	Severe respiratory involvement	Thrombo-damage/redox score	60	29	AUC 0.90; OR/SD 10.30	Score-level association; requires external validation
Composite score	Severe respiratory involvement	Combined multi-axis score	60	29	AUC 0.96; OR/SD 35.60	Score-level association; requires external validation

All models are internally cross-validated and exploratory; performance estimates are event-limited and require external validation. AUC, area under the ROC curve; CI, confidence interval; PLS-DA, partial least squares discriminant analysis.

## Data Availability

The original contributions presented in this study are included in the article/Supplementary Materials. Further inquiries can be directed to the corresponding author(s).

## Supplementary Materials

The following supporting information can be downloaded at: https://www.mdpi.com/article/10.3390/jcm15176725/s1, Figure S1: Variable availability in the hospitalized COVID-19 cohort; Figure S2: Biomarker association ranking by Kruskal–Wallis epsilon-squared effect size; Figure S3: ROC curves for severe respiratory involvement; Figure S4: ROC curves for mortality; Figure S5: Analytical workflow distinguishing admission markers from study-sample biomarkers and summarizing the sequence of descriptive, inferential, ROC, modelling, score and clustering analyses.; Figure S6: Proposed multi-axis model for adverse evolution in hospitalized COVID-19; Figure S7: Context-dependent conceptual interpretation of published differences in soluble IFNAR2; Figure S8: AUC ranking for mortality-associated biomarkers: Figure S9: Exploratory distribution of serum sIFNAR2 in healthy volunteers and hospitalized COVID-19 patients; Figure S10: Hierarchical Spearman correlation matrix of selected analytes; Figure S11: Distribution of risk-oriented module scores by final clinical status; Figure S12: Patient-level heatmap by risk-oriented analytes; Figure S13: Bootstrap stability of LASSO-selected variables for final severity; Figure S14: Decision-curve analysis for final severity; Figure S15: Decision-curve analysis for subsequent worsening after the study sample; Table S1: Variable availability and analytical role; Table S2: Extended clinical and therapeutic profile by final clinical status, Table S3: Pairwise comparisons of sIFNAR2 including healthy controls; Table S4: Extended biomarker associations with final clinical status; Table S5: Extended biomarker analyses by post-sampling clinical outcomes; Table S6: Extended ROC analyses for clinically relevant outcomes; Table S7: Extended summary of multivariable and penalized model performance; Table S8: Composite score performance across outcomes; Table S9: Correlation-based analyte modules and consensus patient clusters; Table S10: Proposed reduced biomarker panels for future validation; Table S11: Preanalytical and analytical characteristics of biomarker measurements; Table S12: Detailed missingness by variable and clinical status group. Table S13: STROBE reporting checklist for the observational cohort study; Table S14: Logistic regression models with odds ratios for selected biomarkers; Table S15: Clinical timing and critical-care characteristics of the study cohort.
