# Supplementary material for: Integrated Inflammatory, Thrombo-Inflammatory, Redox and Soluble IFNAR2 Profiling During Hospitalization for COVID-19: An Exploratory Observational Cohort Study
Source: J Clin Med. 2026 Aug 29;15(17):6725. doi: 10.3390/jcm15176725 (PMC13566477; doi:10.3390/jcm15176725)
Supplement: Supplementary file 1 [file jcm-15-06725-s001.zip › jcm-4461089-supplementary.pdf]

1. Supplementary Material

This supplementary material provides expanded statistical transparency, including variable availability, full biomarker rankings, binary outcome analyses, ROC outputs, model performance, composite scores, molecular modules, clustering and sensitivity-oriented figures. Healthy controls were recruited for the sIFNAR2 comparison; Healthy controls were not included in prognostic models. Composite scores were calculated as risk-oriented standardized summaries of prespecified variables; right-skewed variables were log(1+x)-transformed when appropriate, protective variables were reversed, and variables with substantial missingness were interpreted cautiously. Additional reporting transparency is provided in Supplementary Tables S11-S15.

1.1. Supplementary Tables

Table S1. Variable availability and analytical role.

Variable/domain	Unit or coding	N valid	Missing	% valid	Analytical role
Age	years	60	0	100.0	Admission clinical covariate
Sex	0=male; 1=female	60	0	100.0	Clinical covariate
Final clinical status	favorable; severe; deceased	60	0	100.0	Primary ordinal outcome
Severe respiratory involvement	0=no; 1=yes	60	0	100.0	Main binary outcome
Worsening after study sample	0=no; 1=yes	60	0	100.0	Prospective post-sample outcome
Death	0=no; 1=yes	60	0	100.0	Mortality outcome
Days from symptoms to sample	days	60	0	100.0	Timing variable
Leukocytes at admission	cells/μL	54	6	90.0	Early admission panel
Lymphocytes at admission	cells/μL	54	6	90.0	Early admission panel
CRP at admission	mg/L	56	4	93.3	Early admission panel
LDH at admission	U/L	53	7	88.3	Early admission panel
D-dimer at admission	ng/mL	55	5	91.7	Early admission panel
Ferritin at admission	ng/mL	28	32	46.7	Early admission panel; limited by missingness
sIFNAR2	ng/mL	53	7	88.3	Exploratory interferon-axis marker
IL-6	pg/mL	56	4	93.3	Inflammatory cytokine
IL-10	pg/mL	58	2	96.7	Immunoregulatory cytokine
IL-1RA	pg/mL	48	12	80.0	Counter-regulatory IL-1 pathway marker
Total thiols	μM	55	5	91.7	Redox/oxidative stress marker
Ferritin, study sample	ng/mL	40	20	66.7	Inflammation/tissue-damage marker
D-dimer, study sample	ng/mL	52	8	86.7	Thrombo-inflammatory marker
LDH, study sample	U/L	57	3	95.0	Tissue-damage marker
RAGE	ng/L	45	15	75.0	Endothelial/AGE-RAGE axis
MMP-9	μg/L	56	4	93.3	Matrix remodeling marker
IFN-gamma	pg/mL	36	24	60.0	Type II IFN axis; limited by missingness
TNF-alpha	pg/mL	60	0	100.0	Pro-inflammatory cytokine

N valid, number of non-missing observations; % valid, proportion of the full cohort with available data.

Table S2. Extended clinical and therapeutic profile by final clinical status.

Variable	Favorable outcome (n=31)	Severe non-fatal (n=22)	Deceased (n=7)	p value	Effect size
Age, years	64.0 [56.5-72.5]	64.0 [61.2-71.8]	71.0 [59.0-75.0]	0.681	epsilon²=0.00
Days from symptom onset to study sample	5.00 [3.00-9.50]	7.00 [4.50-8.00]	7.00 [6.00-8.50]	0.766	epsilon²=0.00
Male sex	17/31 (54.8%)	18/22 (81.8%)	5/7 (71.4%)	0.117	V=0.27
Unfavorable status at sampling	1/31 (3.2%)	10/22 (45.5%)	3/7 (42.9%)	0.000704	V=0.49
Worsening after study sample	1/31 (3.2%)	14/22 (63.6%)	7/7 (100.0%)	4.33×10^{-8}	V=0.75
Severe respiratory involvement	2/31 (6.5%)	20/22 (90.9%)	7/7 (100.0%)	1.51×10^{-10}	V=0.87
Death	0/31 (0.0%)	0/22 (0.0%)	7/7 (100.0%)	9.36×10^{-14}	V=1.00
Corticosteroids before sample	9/31 (29.0%)	5/22 (22.7%)	1/7 (14.3%)	0.685	V=0.11
Tocilizumab before sample	2/26 (7.7%)	7/18 (38.9%)	3/4 (75.0%)	0.003	V=0.49
Interferon before sample	2/26 (7.7%)	7/18 (38.9%)	3/4 (75.0%)	0.003	V=0.49

Continuous variables are median [interquartile range, IQR]; categorical variables n (%). ε^2, epsilon-squared (Kruskal–Wallis); V, Cramér's V (categorical).

Table S3. Pairwise comparisons of sIFNAR2 including healthy controls.

Comparison	n1	n2	Median group 1	Median group 2	Median difference	Rank-biserial delta	p value	Holm-adjusted p
Healthy control vs Favorable outcome	12	27	4.20	8.08	3.88	0.81	6.29×10^{-5}	0.000315
Healthy control vs Severe non-fatal	12	19	4.20	8.08	3.88	0.95	1.3×10^{-5}	7.81×10^{-5}
Healthy control vs Deceased	12	7	4.20	17.9	13.7	1.00	0.000453	0.0018
Favorable outcome vs Severe non-fatal	27	19	8.08	8.08	0.00	0.15	0.390	0.390
Favorable outcome vs Deceased	27	7	8.08	17.9	9.85	0.69	0.0060	0.018
Severe non-fatal vs Deceased	19	7	8.08	17.9	9.85	0.59	0.026	0.052

Pairwise Mann–Whitney U comparisons with Holm adjustment. sIFNAR2, soluble interferon-α/β receptor subunit 2; IQR, interquartile range.

Table S4. Extended biomarker associations with final clinical status.

Biomarker	Unit	N	Favorable median [IQR]	Severe median [IQR]	Deceased median [IQR]	p value	FDR q	Effect size	Ordinal rho
IL-6	pg/mL	56	12.0 [10.0-17.0]	70.5 [31.6-164]	36.0 [29.0-87.0]	1.6×10^{-6}	2.71×10^{-5}	epsilon²=0.47	0.67
D-dimer, study sample	ng/mL	52	711 [358-1,312]	3,472 [1,484-4,072]	2,690 [1,904-3,667]	1.84×10^{-5}	0.000156	epsilon²=0.40	0.62
Total thiols	µM	55	275 [246-342]	227 [201-243]	167 [127-210]	4.19×10^{-5}	0.000237	epsilon²=0.35	-0.61
IL-10	pg/mL	58	1.75 [1.54-2.31]	2.20 [1.97-2.95]	3.13 [2.85-3.65]	0.000356	0.002	epsilon²=0.25	0.51
LDH at admission	U/L	53	268 [235-310]	442 [314-642]	328 [310-396]	0.000400	0.002	epsilon²=0.27	0.48
Ferritin, study sample	ng/mL	40	380 [224-581]	814 [671-1,437]	1,608 [1,313-3,224]	0.000418	0.002	epsilon²=0.37	0.63
Leukocytes, study sample	cells/µL	53	6,600[4,890-8,420]	11,110 [7,315-13,330]	10,930 [9,270-12,630]	0.001	0.003	epsilon²=0.23	0.51
LDH, study sample	U/L	57	241 [212-288]	312 [258-374]	394 [298-452]	0.001	0.003	epsilon²=0.21	0.49
IL-1RA	pg/mL	48	640 [224-1,699]	1,197 [1,009-2,168]	5,173 [2,635-7,547]	0.003	0.008	epsilon²=0.22	0.48
CRP at admission	mg/L	56	58.0 [35.5-108]	135 [68.0-256]	68.0 [53.0-122]	0.011	0.026	epsilon²=0.13	0.30
RAGE	ng/L	45	386 [247-741]	179 [74.0-300]	154 [34.8-321]	0.012	0.026	epsilon²=0.16	-0.44
MMP-9	µg/L	56	168 [69.5-228]	230 [213-267]	159 [150-201]	0.017	0.031	epsilon²=0.12	0.26
sIFNAR2	ng/mL	53	8.08 [5.41-10.4]	8.08 [7.08-10.3]	17.9 [9.64-20.2]	0.017	0.031	epsilon²=0.12	0.33

Lymphocytes,study sample	cells/µL	55	1,620 [1,130-1,950]	1,030 [842-1,352]	940 [622-1,400]	0.044	0.070	epsilon^2=0.08	-0.34
Ferritin at admission	ng/mL	28	479 [263-767]	959 [896-1,286]	1,608 [1,044-2,260]	0.048	0.072	epsilon^2=0.16	0.47
TNF-alpha	pg/mL	60	8.00 [8.00-9.00]	9.00 [8.00-10.0]	10.0 [9.00-11.5]	0.067	0.094	epsilon^2=0.06	0.30
TGF-beta	pg/mL	55	494 [357-640]	484 [413-690]	374 [309-454]	0.103	0.137	epsilon^2=0.05	-0.11

Median [IQR] per group. p, Kruskal–Wallis; FDR q, Benjamini–Hochberg adjusted; ε^2, epsilon-squared; ordinal ρ, Spearman correlation with ordinal status. Rows are ordered by nominal p value.

Table S5. Extended biomarker analyses by post-sampling clinical outcomes.

Outcome	Biomarker	Unit	N no	N yes	Median no	Median yes	Cliff delta	p value	FDR q
Severe respiratory involvement	IL-6	pg/mL	31	25	12.0	58.0	0.77	9.64×10^{-7}	7.76×10^{-6}
Severe respiratory involvement	D-dimer, study sample	ng/mL	26	26	711	3,190	0.78	1.29×10^{-6}	7.76×10^{-6}
Severe respiratory involvement	LDH at admission	U/L	26	27	264	416	0.73	5.45×10^{-6}	2.18×10^{-5}
Severe respiratory involvement	Total thiols	µM	30	25	272	212	-0.70	9.12×10^{-6}	2.74×10^{-5}
Severe respiratory involvement	RAGE	ng/L	24	21	408	163	-0.73	3.29×10^{-5}	7.9×10^{-5}
Severe respiratory involvement	Leukocytes, study sample	cells/µL	28	25	6,355	10,950	0.65	5.04×10^{-5}	0.000101
Severe respiratory involvement	Ferritin, study sample	ng/mL	17	23	371	1,046	0.73	9.14×10^{-5}	0.000157
Severe respiratory involvement	CRP at admission	mg/L	28	28	58.0	122	0.52	0.000852	0.003
Severe respiratory involvement	IL-10	pg/mL	31	27	1.81	2.49	0.50	0.001	0.003
Severe respiratory involvement	IL-1RA	pg/mL	23	25	608	1,732	0.54	0.001	0.003
Severe respiratory involvement	Ferritin at admission	ng/mL	14	14	426	1,040	0.66	0.003	0.006
Severe respiratory involvement	LDH, study sample	U/L	31	26	245	313	0.46	0.003	0.006
Subsequent worsening	IL-6	pg/mL	37	19	13.0	38.0	0.55	0.000884	0.012
Subsequent worsening	Total thiols	µM	37	18	266	216	-0.55	0.000997	0.012
Subsequent worsening	IL-1RA	pg/mL	28	20	748	2,075	0.50	0.003	0.027
Subsequent worsening	Lymphocytes, study sample	cells/µL	35	20	1,580	965	-0.44	0.008	0.046
Subsequent worsening	Leukocytes, study sample	cells/µL	36	17	7,190	10,930	0.39	0.025	0.103
Subsequent worsening	Ferritin, study sample	ng/mL	24	16	563	1,090	0.41	0.030	0.103
Subsequent worsening	D-dimer, study sample	ng/mL	33	19	1,065	2,396	0.35	0.038	0.103
Subsequent worsening	TNF-alpha	pg/mL	38	22	8.00	10.0	0.31	0.039	0.103
Subsequent worsening	Ferritin at admission	ng/mL	16	12	569	1,136	0.47	0.039	0.103
Subsequent worsening	IL-10	pg/mL	38	20	1.94	2.40	0.32	0.048	0.114
Subsequent worsening	RAGE	ng/L	30	15	342	196	-0.33	0.077	0.166
Subsequent worsening	LDH at admission	U/L	33	20	283	332	0.28	0.090	0.166

Death	Total thiols	µM	49	6	246	167	-0.76	0.001	0.019
Death	IL-10	pg/mL	52	6	1.97	3.13	0.77	0.002	0.019
Death	Ferritin, study sample	ng/mL	35	5	646	1,608	0.79	0.002	0.019
Death	IL-1RA	pg/mL	41	7	1,115	5,173	0.70	0.003	0.020
Death	sIFNAR2	ng/mL	46	7	8.08	17.9	0.65	0.007	0.031
Death	LDH, study sample	U/L	50	7	260	394	0.53	0.026	0.089
Death	TGF-beta	pg/mL	48	7	484	374	-0.48	0.043	0.130
Death	Leukocytes, study sample	cells/µL	48	5	7,715	10,930	0.47	0.091	0.241
Death	CRP, study sample	mg/L	53	7	20.0	90.0	0.38	0.107	0.241
Death	IL-6	pg/mL	51	5	17.0	36.0	0.44	0.111	0.241
Death	D-dimer, study sample	ng/mL	46	6	1,358	2,690	0.38	0.135	0.248

Median values per outcome group. Cliff's delta, non-parametric effect size; p, Mann–Whitney U; FDR q, Benjamini–Hochberg adjusted value. Exploratory, event-limited.

Table S6. Extended ROC analyses for clinically relevant outcomes.

Outcome	Marker	Unit	N	Events	AUC	95% CI	Risk direction	Youden cut-off	Sensitivity	Specificity
Severe respiratory involvement	D-dimer, study sample	ng/mL	52	26	0.89	0.79-0.96	higher values indicate higher risk	>=1,355	0.92	0.77
Severe respiratory involvement	IL-6	pg/mL	56	25	0.88	0.80-0.95	higher values indicate higher risk	>=27.0	0.80	0.84
Severe respiratory involvement	Ferritin, study sample	ng/mL	40	23	0.87	0.74-0.97	higher values indicate higher risk	>=646	0.87	0.82
Severe respiratory involvement	Leukocytes, study sample	cells/µL	53	25	0.83	0.71-0.92	higher values indicate higher risk	>=8,960	0.72	0.82
Severe respiratory involvement	IL-10	pg/mL	58	27	0.75	0.61-0.86	higher values indicate higher risk	>=2.43	0.59	0.87
Severe respiratory involvement	IL-1RA	pg/mL	48	25	0.77	0.63-0.89	higher values indicate higher risk	>=985	0.84	0.65
Severe respiratory involvement	LDH, study sample	U/L	57	26	0.73	0.57-0.85	higher values indicate higher risk	>=339	0.46	0.94
Severe respiratory involvement	sIFNAR2	ng/mL	53	26	0.69	0.54-0.81	higher values indicate higher risk	>=10.9	0.38	0.93
Subsequent worsening	IL-6	pg/mL	56	19	0.77	0.65-0.89	higher values indicate higher risk	>=14.0	1.00	0.51
Subsequent worsening	IL-1RA	pg/mL	48	20	0.75	0.60-0.86	higher values indicate higher risk	>=1,181	0.75	0.64
Subsequent worsening	Lymphocytes, study sample	cells/µL	55	20	0.72	0.56-0.86	lower values indicate higher risk	<=1,450	0.85	0.60
Subsequent worsening	Ferritin, study sample	ng/mL	40	16	0.71	0.50-0.87	higher values indicate higher risk	>=696	0.81	0.62
Subsequent worsening	D-dimer, study sample	ng/mL	52	19	0.67	0.53-0.82	higher values indicate higher risk	>=890	1.00	0.45
Subsequent worsening	IL-10	pg/mL	58	20	0.66	0.52-0.80	higher values indicate higher risk	>=2.20	0.70	0.61

Subsequent worsening	sIFNAR2	ng/mL	53	19	0.64	0.48-0.77	higher values indicate higher risk	>=7.29	0.84	0.44
Death	Ferritin, study sample	ng/mL	40	5	0.90	0.78-1.00	higher values indicate higher risk	>=1,164	1.00	0.83
Death	IL-10	pg/mL	58	6	0.88	0.78-0.98	higher values indicate higher risk	>=2.43	1.00	0.73
Death	IL-1RA	pg/mL	48	7	0.85	0.67-0.98	higher values indicate higher risk	>=2,126	0.86	0.78
Death	sIFNAR2	ng/mL	53	7	0.82	0.64-0.97	higher values indicate higher risk	>=10.9	0.71	0.85
Death	LDH, study sample	U/L	57	7	0.76	0.49-0.92	higher values indicate higher risk	>=339	0.71	0.82

AUC, area under the ROC curve (95% confidence interval); sens., sensitivity; spec., specificity. Youden-based cut-offs are indicative only.

Table S7. Extended summary of multivariable and penalized model performance.

Outcome	Panel/model	N	Events	Performance metric	Value	Interpretation
Severe/death vs favorable outcome	Admission panel	60	29	Mean CV AUC	0.74 (0.69-0.77)	Internal cross-validation; exploratory
Severe/death vs favorable outcome	Biological panel	60	29	Mean CV AUC	0.89 (0.88-0.91)	Internal cross-validation; exploratory
Severe/death vs favorable outcome	Combined panel	60	29	Mean CV AUC	0.89 (0.84-0.92)	Internal cross-validation; exploratory
Severe respiratory involvement	Admission panel	60	29	Mean CV AUC	0.86 (0.82-0.88)	Internal cross-validation; exploratory
Severe respiratory involvement	Biological panel	60	29	Mean CV AUC	0.94 (0.91-0.95)	Internal cross-validation; exploratory
Severe respiratory involvement	Combined panel	60	29	Mean CV AUC	0.97 (0.97-0.98)	Internal cross-validation; exploratory
Subsequent worsening	Admission panel	60	22	Mean CV AUC	0.51 (0.45-0.56)	Internal cross-validation; exploratory
Subsequent worsening	Biological panel	60	22	Mean CV AUC	0.69 (0.62-0.74)	Internal cross-validation; exploratory
Subsequent worsening	Combined panel	60	22	Mean CV AUC	0.70 (0.63-0.76)	Internal cross-validation; exploratory
Death	Admission panel	60	7	Mean CV AUC	0.76 (0.60-0.91)	Internal cross-validation; event-limited for mortality
Death	Biological panel	60	7	Mean CV AUC	0.89 (0.85-0.92)	Internal cross-validation; event-limited for mortality
Death	Combined panel	60	7	Mean CV AUC	0.95 (0.88-1.00)	Internal cross-validation; event-limited for mortality

All models internally cross-validated and exploratory. AUC, area under the ROC curve; CI, confidence interval.

Table S8. Composite score performance across outcomes.

Outcome	Composite score	N	Events	AUC	OR per SD (95% CI)	Mann-Whitney p	Logistic p
Severe/death vs favorable outcome	Early admission score	60	29	0.76	3.00 (1.54-5.85)	0.001	0.001
Severe/death vs favorable outcome	Biological-immune score	60	29	0.91	15.10 (4.00-57.04)	0.001	0.001
Severe/death vs favorable outcome	Thrombo-damage/redox score	60	29	0.89	12.20 (3.52-42.31)	0.001	0.001
Severe/death vs favorable outcome	Combined multi-axis score	60	29	0.94	26.22 (4.85-141.70)	0.001	0.001
Severe respiratory involvement	Early admission score	60	29	0.80	4.10 (1.91-8.80)	0.001	0.001
Severe respiratory involvement	Biological-immune score	60	29	0.93	12.90 (3.91-42.61)	0.001	0.001
Severe respiratory involvement	Thrombo-damage/redox score	60	29	0.90	10.30 (3.34-31.75)	0.001	0.001
Severe respiratory involvement	Combined multi-axis score	60	29	0.96	35.60 (5.73-221.20)	0.001	0.001
Subsequent worsening	Early admission score	60	22	0.66	1.90 (1.03-3.50)	0.038	0.041
Subsequent worsening	Biological-immune score	60	22	0.76	3.10 (1.42-6.78)	0.003	0.004
Death	Biological-immune score	60	7	0.89	6.40 (1.71-23.90)	0.004	0.006
Death	Combined multi-axis score	60	7	0.95	11.20 (1.76-71.30)	0.001	0.011

OR per SD, odds ratio per standard-deviation increase; AUC, area under the ROC curve; CI, confidence interval. Exploratory.

Table S9. Correlation-based analyte modules and consensus patient clusters.

Analysis	Group/module	Markers or phenotype	Key clinical interpretation
Analyte module	Dominant inflammation-thrombo-damage/redox module	IL-6, D-dimer, total thiols, RAGE, leukocytes, ferritin, LDH, IL-10, IL-1RA, MMP-9, lymphocytes, TNF-alpha	Association with ordinal severity in this cohort
Analyte module	sIFNAR2 module	sIFNAR2	Partly independent interferon receptor-axis signal
Patient cluster	Cluster 1 (n=31)	27 favorable, 4 severe, 0 deaths; distress 3/31, worsening 5/31	Low-risk phenotype
Patient cluster	Cluster 2 (n=25)	4 favorable, 15 severe, 6 deaths; distress 22/25, worsening 13/25	Exploratory cluster enriched for adverse outcomes
Patient cluster	Cluster 3 (n=4)	0 favorable, 3 severe, 1 deaths; distress 4/4, worsening 4/4	Exploratory high-inflammatory cluster (n=4)

Modules derived from Spearman correlation structure; consensus clusters are descriptive and hypothesis-generating.

Table S10. Proposed reduced biomarker panels for future validation.

Clinical question	Candidate reduced panel	Rationale	Recommended sampling time	Validation priority
Early emergency triage	LDH, CRP, ferritin, D-dimer, lymphocyte count	Accessible and available at hospital admission	Emergency department/admission	High
Respiratory deterioration risk	IL-6, D-dimer, ferritin, leukocytes, IL-1RA	Best distress-related signals in this cohort	Admission plus days 3-5 and 7-10	High
Post-sample worsening	IL-6, IL-1RA, lymphocytes, ferritin, D-dimer	Biological signal stronger than admission-only models	During hospitalization; repeat if clinical change	Moderate-high
Mortality subphenotype	IL-10, IL-1RA, ferritin, LDH, sIFNAR2	Mortality-associated immune-regulatory and tissue-damage pattern	During hospitalization; ideally longitudinal	High but event-limited

Interferon-axis biology	sIFNAR2, IFNAR1/2 genotype, anti-type I IFN autoantibodies, IFN-stimulated gene signature	Needed to interpret context-dependent sIFNAR2 findings	Prospective serial serum/plasma with standardized matrix	Very high
Redox/injury axis	Total thiols, LDH, ferritin, RAGE/MMP-9	Links oxidative stress, tissue injury and endothelial/matrix biology	Admission and serial study samples	Moderate

Reduced panels are proposals for external validation and are not validated prediction tools.

Table S11. Preanalytical and analytical characteristics of biomarker measurements.

Domain / analyte group	Matrix and method	Units / valid observations	Preanalytical and analytical notes
Soluble IFNAR2 (sIFNAR2)	Serum; commercially available colorimetric sandwich ELISA for solu-ble IFNAR2 (Human IF-NAR2 ELISA Kit, Abcam, Cat# ab264610); optical density read at 450 nm; 96-well pre-coated micro-plate; samples diluted 1:8 and final concentrations corrected for dilution.	ng/mL; valid values: 53/60 hospitalized patients and 12/18 healthy controls. Manufacturer-reported calibration range: 46.875-3000 pg/mL; analytical sensitivity: 3.72 pg/mL; serum intra-assay CV: 5.0%; serum inter-assay CV: 2.3%; serum recovery: 100% (96-109%).	Used as the prespecified soluble IFNAR2 measurement. Healthy controls were used only for sIFNAR2 comparison and not for prognostic models. Values below the detec-tion/quantification limit were not extrapolated beyond the standard curve and were excluded from quantitative analysis. Detailed plate/batch allocation and lot-level traceability were not available in the analytical dataset.
Cytokine / immune panel: IL-6, IL-10, IL-1RA, IL-1beta, IL-12, IL-17, IFN-beta, TNF-alpha, TNF-beta, IFN-gamma, IL-13, IL-4	Study serum sample; multiplex immunoassay platform as recorded in the laboratory dataset.	Mostly pg/mL; individual valid observations are reported in Supplementary Table S1 and missingness in Supplementary Table S12.	Several analytes showed substantial missingness or low detectability and were therefore interpreted cautiously.
Redox / oxidative stress marker: total thiols	Study serum sample; biochemical redox assay as used in the original cohort analyses.	μM; valid values: 55/60 hospitalized patients.	Interpreted as a systemic antioxidant-reserve marker.
Routine admission markers: CRP, LDH, D-dimer, ferritin, leukocytes and lymphocytes	Hospital admission blood tests; routine clinical laboratory methods.	Units as shown in Supplementary Tables S1, S4-S6 and S12.	These markers provide the most clinically transferable component of the analysis.
Study-sample routine / tissue-damage and thrombo-inflammatory markers: ferritin, D-dimer, LDH, leukocytes, lymphocytes and CRP	Study blood sample during hospitalization; routine clinical laboratory methods.	Units as shown in Supplementary Tables S1, S4-S6 and S12.	These variables were interpreted primarily as markers of biological state during established admission and, when temporally appropriate, as predictors of subsequent course. They are not presented as pure emergency-department triage markers.

Endothelial / matrix-remodeling markers: RAGE and MMP-9	Study serum sample; immunoassay-based measurement according to the original laboratory workflow.	RAGE, ng/L; MMP-9, μg/L; valid observations reported in Supplementary Tables S1 and S12.	Interpreted as exploratory endothelial/AGE-RAGE and matrix-remodeling markers.

Summary of matrix, assay method and preanalytical handling per analyte group.

Table S12. Detailed missingness by variable and clinical status group.

Variable	N valid total	Missing total	Missing favorable (n=31)	Missing severe non-fatal (n=22)	Missing deceased (n=7)
Age	60	0	0/31	0/22	0/7
Sex	60	0	0/31	0/22	0/7
Days from symptoms to sample	60	0	0/31	0/22	0/7
Leukocytes at admission	54	6	3/31	1/22	2/7
Lymphocytes at admission	54	6	3/31	1/22	2/7
CRP at admission	56	4	3/31	1/22	0/7
LDH at admission	53	7	5/31	2/22	0/7
D-dimer at admission	55	5	3/31	1/22	1/7
Ferritin at admission	28	32	17/31	11/22	4/7
sIFNAR2	53	7	4/31	3/22	0/7
IL-6	56	4	0/31	2/22	2/7
3IL-10	58	2	0/31	1/22	1/7
IL-1RA	48	12	8/31	4/22	0/7
Total thiols	55	5	2/31	2/22	1/7
Ferritin, study sample	40	20	15/31	3/22	2/7
D-dimer, study sample	52	8	5/31	2/22	1/7
LDH, study sample	57	3	0/31	3/22	0/7
RAGE	45	15	7/31	5/22	3/7
MMP-9	56	4	1/31	2/22	1/7
IFN-gamma	36	24	16/31	7/22	1/7
TNF-alpha	60	0	0/31	0/22	0/7
TGF-beta	55	5	3/31	2/22	0/7
Leukocytes, study sample	53	7	2/31	3/22	2/7
Lymphocytes, study sample	55	5	2/31	2/22	1/7
CRP, study sample	60	0	0/31	0/22	0/7

Missingness shown overall and per clinical status group. Analytes with substantial missingness were interpreted with caution.

Table S13. STROBE reporting checklist for the observational cohort study.
This table is aligned with the final manuscript and supplementary material. It documents reporting completeness for an exploratory observational cohort and does not imply confirmatory prediction-model validation.

STROBE item	Reporting element	Location in manuscript/supplement	Status / comments
1. Title and abstract	Indicate study design and provide an informative abstract.	Title; Abstract.	Reported. The title and abstract identify the work as an exploratory observational cohort study. The abstract reports the hospitalized COVID-19 cohort, healthy controls for sIFNAR2 comparison, outcomes and analytical methods.
2. Background/rationale	Explain the scientific background and rationale.	Introduction.	Reported. The scientific rationale is framed around integrated inflammatory, thrombo-inflammatory, tissue-damage, redox and interferon-axis biology in severe COVID-19.
3. Objectives	State specific objectives and hypotheses.	End of Introduction.	Reported. The objectives are exploratory, mechanistic and prognostic: to evaluate whether adverse clinical evolution is associated with a coordinated multi-axis biomarker profile and to define the added value and limitations of sIFNAR2.
4. Study design	Present key elements of study design early in the paper.	Materials and Methods: Study Design and Participants.	Reported. The study is described as an exploratory observational analysis of a single-center first-wave hospitalized COVID-19 cohort and as an extension of a previously published work in the same cohort.
5. Setting	Describe setting, locations, recruitment dates and follow-up.	Materials and Methods: Study Design and Participants; affiliations; Institutional Review Board Statement.	Reported. The study was conducted in a single-center hospitalized COVID-19 cohort from the first pandemic wave in 2020, linked to Hospital Universitario Virgen de la Victoria/IBIMA-BIONAND, Malaga, Spain. Ethical approval was granted by the Ethics Committee of Malaga University Hospital, protocol code 01-2020, approval date 7 May 2020.
6. Participants	Describe eligibility criteria, sources and methods of selection of participants.	Materials and Methods: Study Design and Participants; Supplementary Table S1.	Reported. Eligible participants were adults aged 18 years or older with SARS-CoV-2 infection confirmed by rRT-PCR using the cobas test from nasopharyngeal swab, sputum or bronchoalveolar lavage, together with pneumonia or severe respiratory involvement. Asymptomatic RT-PCR-positive patients and those without radiological findings compatible with pneumonia or severe respiratory involvement were excluded. Healthy controls were recruited for sIFNAR2 comparison; controls were not included in prognostic models.
7. Variables	Clearly define outcomes, exposures, predictors, potential confounders and effect modifiers.	Materials and Methods: Biomarker panel; Clinical outcomes and temporal framework; Supplementary Tables S1, S11 and S12.	Reported. Outcomes comprise final clinical status, severe/death versus favorable outcome, severe respiratory involvement, worsening after the study biomarker sample and death. Candidate predictors include admission markers, study-sample biomarkers, cytokine/immune markers, redox and tissue-damage markers, endothelial/matrix markers and sIFNAR2. Pre-sampling corticosteroid, tocilizumab and interferon exposure are reported as treatment-related confounders.

8. Data sources / measurement	For each variable of interest, describe sources of data and details of methods of assessment or measurement.	Materials and Methods: Biomarker panel; Supplementary Table S11.	Reported. Routine clinical markers were obtained from hospital laboratory data. sIFNAR2 was measured in serum using a colorimetric sandwich ELISA, Human IFNAR2 ELISA Kit, Abcam, Cat# ab264610, with optical density read at 450 nm. Cytokines and immune mediators were measured using ProcartaPlex multiplex immunoassay, Thermo Fisher Scientific. Total thiols were measured by spectrophotometry using an ICubio autoanalyzer. D-dimer is reported in ng/mL using DDU calibration.
9. Bias	Describe efforts to address potential sources of bias.	Methods; Discussion; Supplementary Appendix 2.	Reported. Potential bias is addressed through explicit exploratory framing and discussion of temporal ambiguity, single-center first-wave setting, treatment confounding, missingness, limited events, optimistic internal validation and lack of external validation.
10. Study size	Explain how the study size was arrived at.	Materials and Methods; Discussion.	Reported. The sample size corresponds to the available cohort: 60 hospitalized COVID-19 patients, with 18 healthy controls recruited for sIFNAR2 comparison. No formal sample-size calculation was performed because the analysis is exploratory.
11. Quantitative variables	Explain how quantitative variables were handled in the analyses.	Methods: Statistical Analysis; Supplementary Tables S1 and S4-S8.	Reported. Continuous variables are summarized as median [interquartile range] and, where informative, mean +/- standard deviation. Right-skewed variables were log(1+x)-transformed where appropriate and standardized before modelling. Protective variables such as lymphocyte count and total thiols were reversed in risk-oriented composite scores.
12. Statistical methods	Describe all statistical methods, including those used to control for confounding, examine subgroups and interactions, address missing data and perform sensitivity analyses.	Methods: Statistical Analysis; Supplementary Tables S4-S10; Supplementary Appendix 2.	Reported. Analyses included Kruskal-Wallis tests with epsilon-squared, Mann-Whitney U tests with Cliff's delta, chi-square or exact tests with Cramer's V, Benjamini-Hochberg FDR adjustment, Holm-adjusted pairwise sIFNAR2 comparisons, exploratory ROC curves with Youden cut-offs, parsimonious logistic regression, ridge-penalized logistic regression with median imputation, standardization and repeated five-fold cross-validation, composite scores, correlation analysis, clustering, PLS-DA, bootstrap/LASSO stability and decision-curve figures. Calibration metrics and externally validated prediction modelling were not available and are not claimed.
13. Participants / flow	Report numbers of individuals at each stage of the study.	Results: Cohort structure and outcomes; Supplementary Table S1; Supplementary Figure S1.	Reported. The cohort included 60 hospitalized COVID-19 patients. Final clinical status was favorable outcome in 31 patients, severe non-fatal disease in 22 and death in 7. Severe respiratory involvement occurred in 29 patients and worsening after the study sample occurred in 22. Valid sIFNAR2 measurements were available for 53/60 patients and 12/18 healthy controls.
14. Descriptive data	Provide characteristics of study participants and information on missing data.	Table 1; Supplementary Tables S1, S2 and S12.	Reported. Baseline clinical variables, outcome structure, pre-sampling treatment variables and missingness by clinical status group are provided. Missingness is explicitly reported and analytes with substantial missingness are interpreted cautiously.
15. Outcome data	Report numbers of outcome events or summary measures.	Results; Table 1; Supplementary Table S2.	Reported. Outcome data are provided for final clinical status, severe/death status, severe respiratory involvement, worsening after the biomarker sample and death.
16. Main results	Provide unadjusted and adjusted estimates where relevant.	Results; Tables 2-4; Supplementary Tables S3-S10.	Reported. Main results include biomarker associations with final status and post-sampling outcomes, exploratory ROC outputs, parsimonious and penalized models, composite-score performance, patient clusters and reduced panels for future validation. Complex models are interpreted as exploratory and not as validated prediction tools.
17. Other analyses	Report other analyses performed, including subgroup analyses, sensitivity analyses and exploratory analyses.	Supplementary Tables S3-S10; Supplementary Figures S1-S15.	Reported. Additional analyses include pairwise sIFNAR2 comparisons, full biomarker rankings, binary outcome analyses, ROC analyses, model performance, composite scores, correlation-based analyte modules, clustering, proposed reduced panels, bootstrap/LASSO stability and decision-curve figures. These analyses are hypothesis-generating.

18. Key results	Summarize key results with reference to study objectives.	Discussion; Conclusions.	Reported. The discussion emphasizes that conventional inflammatory, thrombo-inflammatory, tissue-damage and redox markers were the most robust signals, whereas sIFNAR2 provided a context-dependent exploratory signal mainly linked to mortality.
19. Limitations	Discuss limitations of the study, taking into account sources of potential bias or imprecision.	Discussion; Supplementary Appendix 2.	Reported. Limitations include the single-center first-wave setting, small sample size, only seven deaths, non-randomized treatment exposure, incomplete treatment timing, temporal ambiguity at sampling, missing analytes, optimistic advanced models and need for sIFNAR2 assay standardization.
20. Interpretation	Provide a cautious overall interpretation of results considering objectives, limitations, multiplicity of analyses and evidence from similar studies.	Discussion and Conclusions.	Reported. Interpretation is conservative: the findings are hypothesis-generating, sIFNAR2 is not presented as a stand-alone test or direct functional measure of IFNAR signaling, and model outputs are not proposed for clinical decision-making.
21. Generalizability	Discuss the generalizability or external validity of the study results.	Discussion and Conclusions.	Reported. External validity is limited by the first-wave, single-center, hospitalized cohort and early-pandemic treatment context. Prospective, longitudinal and multicenter validation with standardized sampling is required before clinical implementation.
22. Funding	Give the source of funding and the role of the funders.	Declarations: Funding.	Reported. The study was funded by Grupo de Investigación de Neumología FIMABIS—Fundación para la Investigación de Málaga en Biomedicina y Salud IBIMA Plataforma BIONAND, Plan Andaluz de Investigación, Desarrollo e Innovación (PAIDI), CTS-156 and Proyectos de Colaboración Público-Privada, Plan Estatal de Investigación Científica, Técnica y de Innovación 2021-2023, grant number CPP2021-008512, Spain. No private company, commercial sponsor or external funder participated in the design of the study, patient recruitment, data collection, laboratory analyses, statistical analysis, interpretation of results, manuscript preparation or decision to submit.

Table S14. Logistic regression models with odds ratios for selected biomarkers.

Outcome	Marker	N	Model	OR per 1-SD increase	95% CI	p value	Interpretation
Severe/death vs favorable outcome	IL-6	56	Univariable	9.54	2.97-30.68	0.000154	Strongest univariable association
Severe/death vs favorable outcome	D-dimer, study sample	52	Univariable	6.95	2.56-18.89	0.000145	Thrombo-inflammatory signal
Severe/death vs favorable outcome	Ferritin, study sample	40	Univariable	4.49	1.50-13.47	0.007	Tissue-damage/inflammatory signal
Severe/death vs favorable outcome	LDH, study sample	57	Univariable	3.38	1.61-7.10	0.0013	Tissue-damage signal
Severe/death vs favorable outcome	sIFNAR2	53	Univariable	2.24	1.14-4.38	0.019	Significant univariable signal
Severe/death vs favorable outcome	IL-6	49	sIFNAR2 + IL-6	9.86	2.58-37.69	0.000821	Retained after adjustment
Severe/death vs favorable outcome	sIFNAR2	49	sIFNAR2 + IL-6	1.89	0.77-4.67	0.166	Not independently retained
Death	sIFNAR2	53	Univariable only	4.01	1.54-10.48	0.005	Strongest stable mortality signal
Death	LDH, study sample	57	Univariable only	3.05	1.20-7.74	0.019	Event-limited mortality signal
Death	Ferritin, study sample	40	Univariable only	16.63	1.58-174.82	0.019	Unstable wide CI; likely overfit
Death	D-dimer, study sample	52	Univariable only	2.43	0.77-7.69	0.132	Not statistically significant
Death	IL-6	56	Univariable only	1.62	0.70-3.77	0.260	Not statistically significant

ORs are per 1-standard-deviation increase in log(1+x)-transformed marker values. For the severe/death composite outcome, sIFNAR2 was evaluated alone and in a two-variable model with IL-6. For mortality, only univariable models were reported because the number of deaths was small (n = 7), making multivariable modelling inappropriate.

Table S15. *Clinical timing and critical-care characteristics of the study cohort.*

A. Clinical timing according to final clinical status

Variable	*Favorable outcome (n = 31)*	*Severe non-fatal (n = 22)*	*Death (n = 7)*
Time from symptom onset to study sample, days	*5.0 [3.0–9.5]*	*7.0 [4.5–8.0]*	*7.0 [6.0–8.5]*

Clinical worsening after study sample, n (%)	*1/31 (3.2)*	*14/22 (63.6)*	*7/7 (100.0)*
Time from study sample to clinical worsening, days	*6 (n = 1)*	*4.0 [2.5–8.0] (n = 11)*	*4.0 [2.5–6.5] (n = 4)*
In-hospital death, n (%)	*0/31 (0.0)*	*0/22 (0.0)*	*7/7 (100.0)*
Time from clinical worsening to death, days	*—*	*—*	*28 [14.5–32.0]*
Onset of study-defined severe respiratory involvement	*Not systematically recorded*	*Not systematically recorded*	*Not systematically recorded*

B. Previously reported critical-care characteristics

Variable	***Value***
ICU admission during hospitalization, n/N (%)	*22/60 (36.7)*
Invasive mechanical ventilation at the time of follow-up biomarker sampling, among the 21 ICU-admitted patients meeting the respiratory severity criteria in the previous report, n/N (%)	*8/21 (38.1)*

Continuous variables are presented as median [interquartile range]. The interval from study sampling to clinical worsening was available for 16 of the 22 patients with post-sampling worsening. Critical-care characteristics are reproduced from the previous report of this cohort [16] and were not retrospectively reclassified according to the final clinical-status categories used in the present analysis. ICU admission refers to hospitalization in the ICU during admission, whereas invasive mechanical ventilation refers specifically to ventilatory support at the time of follow-up biomarker sampling. Respiratory severity in the previous report of this cohort was classified using the criteria applied in that analysis; these figures are reported as published and were not reassessed for the present analysis.

1.2. Supplementary Figures

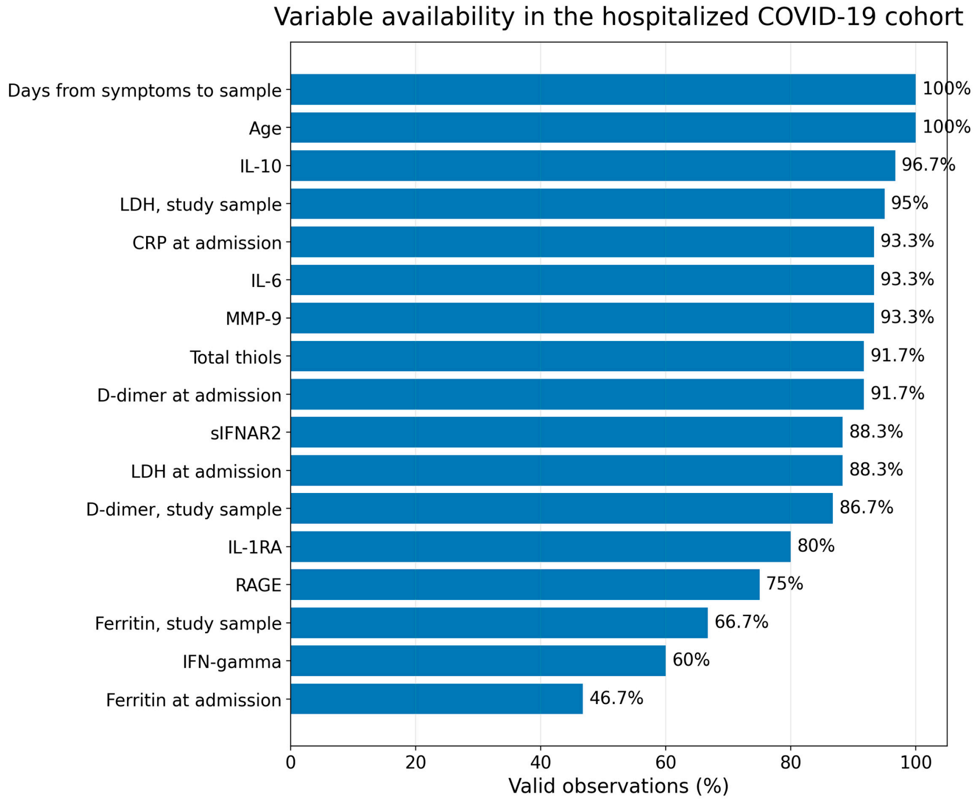

Supplementary Figure S1. Variable availability in the hospitalized COVID-19 cohort. Horizontal bars show the percentage of valid observations for clinical, admission and study-sample biomarkers used in the exploratory analyses.

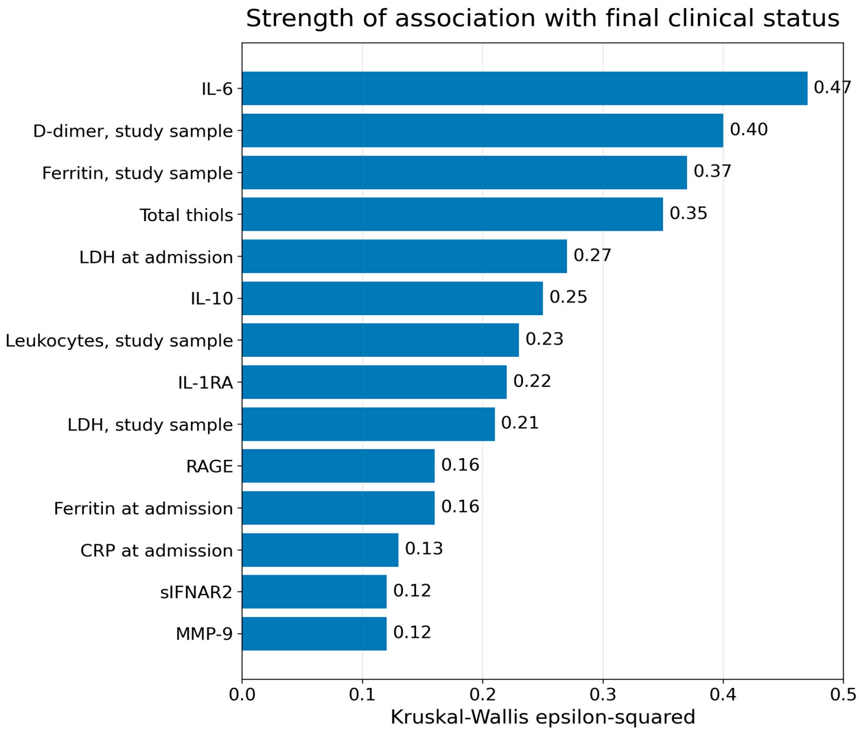

Supplementary Figure S2. Biomarker association ranking by Kruskal-Wallis epsilon-squared effect size. Higher values indicate stronger global association with final clinical status.

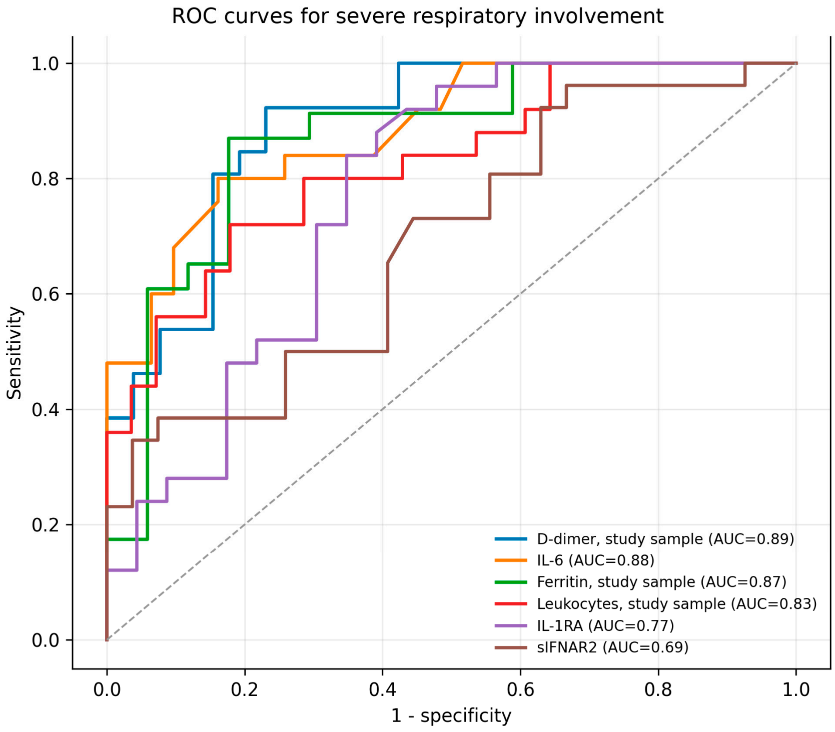

Supplementary Figure S3. ROC curves for severe respiratory involvement. Curves show discrimination for selected biomarkers with the strongest univariable signal.

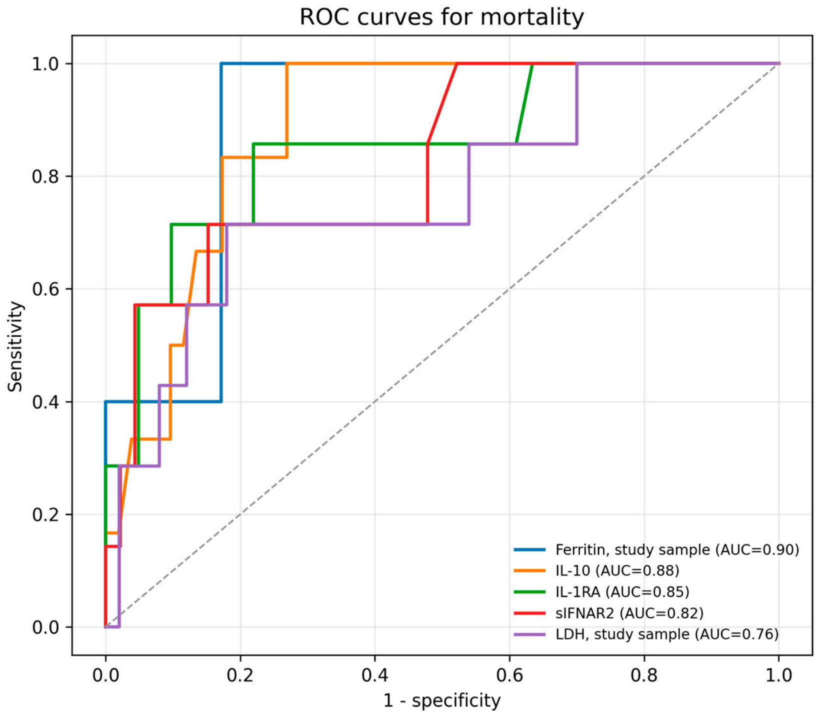

Supplementary Figure S4. ROC curves for mortality. Estimates are exploratory and event-limited because only seven deaths occurred.

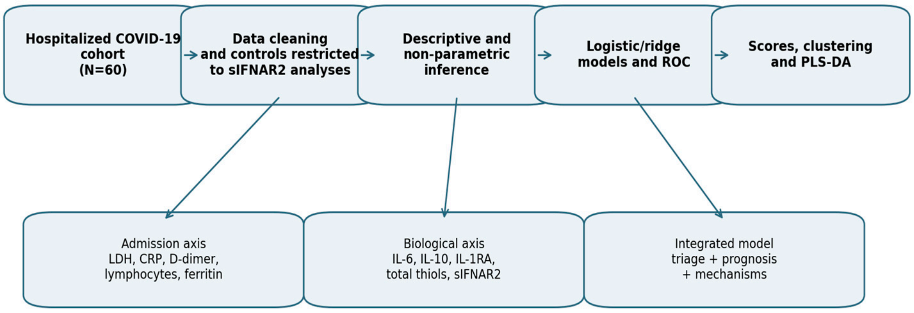

Supplementary Figure S5. Analytical workflow distinguishing admission markers from study-sample biomarkers and summarizing the sequence of descriptive, inferential, ROC, modelling, score and clustering analyses.

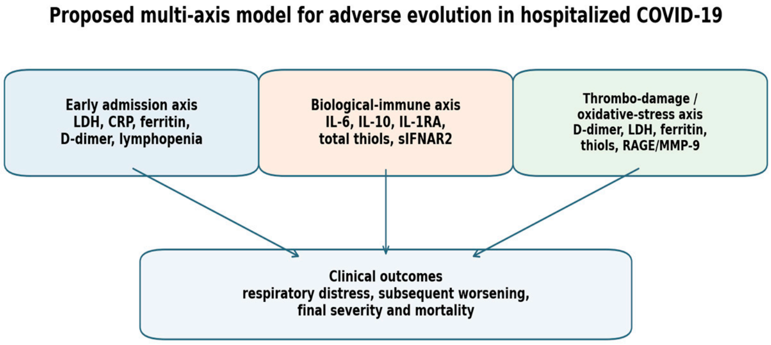

Supplementary Figure S6. Proposed multi-axis model for adverse evolution in hospitalized COVID-19. The model integrates early admission variables, biological-immune markers and thrombo-damage/redox markers as complementary contributors to clinical out

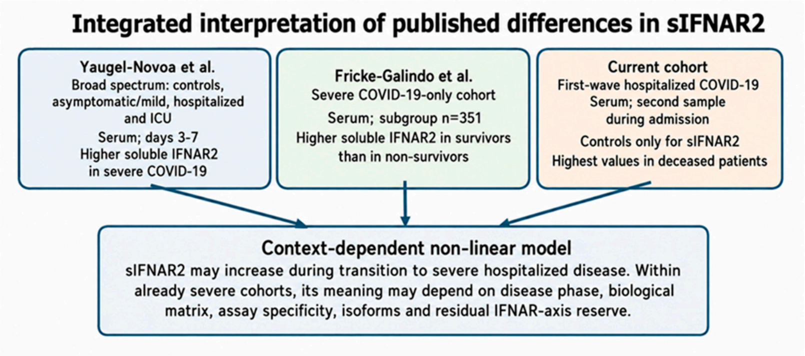

Supplementary Figure S7. Context-dependent conceptual interpretation of published differences in soluble IFNAR2. The diagram summarizes why sIFNAR2 directionality may vary across cohorts according to disease spectrum, biological matrix, sampling time, assay specificity and residual interferon-axis reserve; it should not be interpreted as proof of a validated non-linear biological relationship.

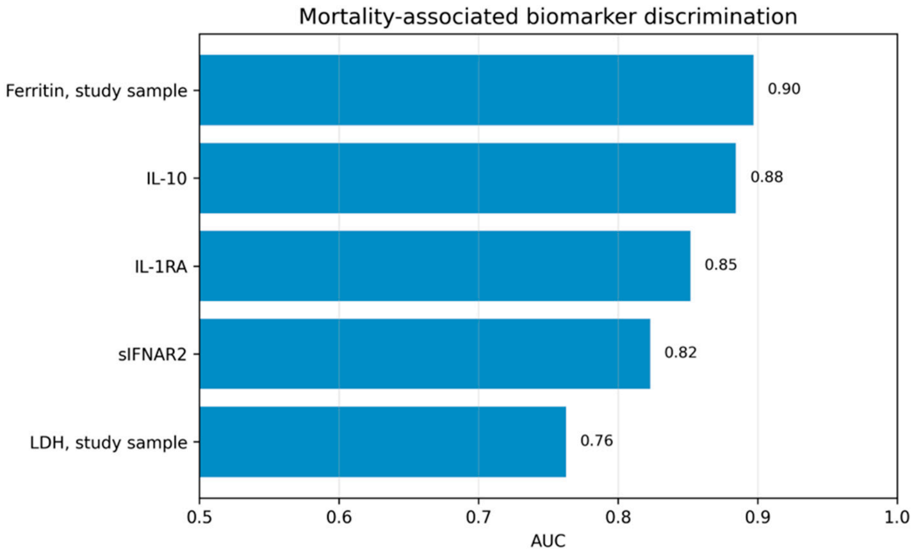

Supplementary Figure S8. AUC ranking for mortality-associated biomarkers. Confidence intervals are shown for selected markers; estimates are exploratory because of the small number of deaths.

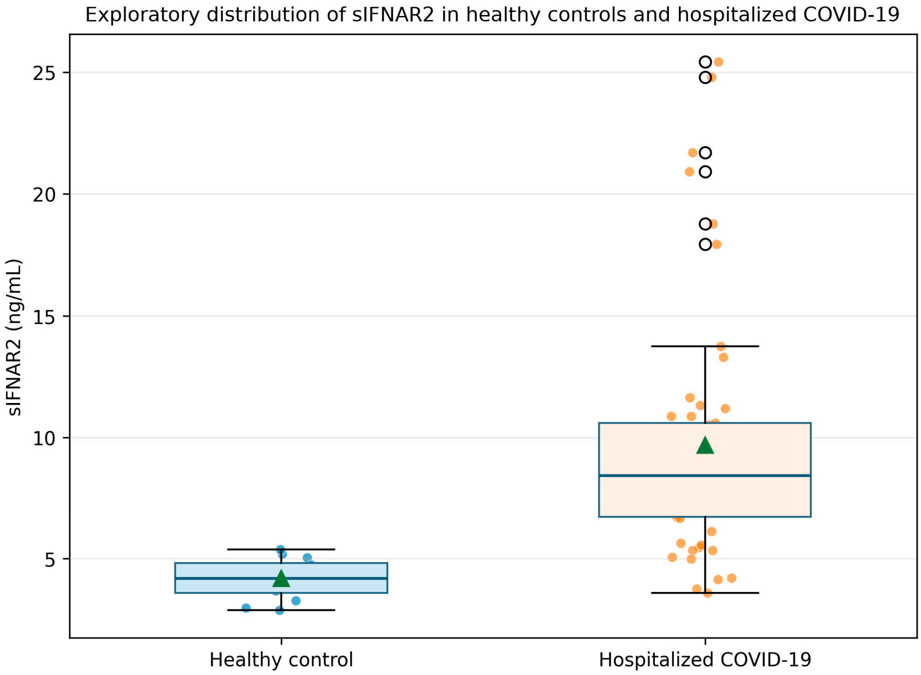

Supplementary Figure S9. Exploratory distribution of serum sIFNAR2 in healthy volunteers and hospitalized COVID-19 patients. Healthy volunteers do not represent a clinically relevant differential-diagnosis population. This comparison is descriptive and exploratory; it is not intended to define, and does not support, any diagnostic test or diagnostic-separation claim.

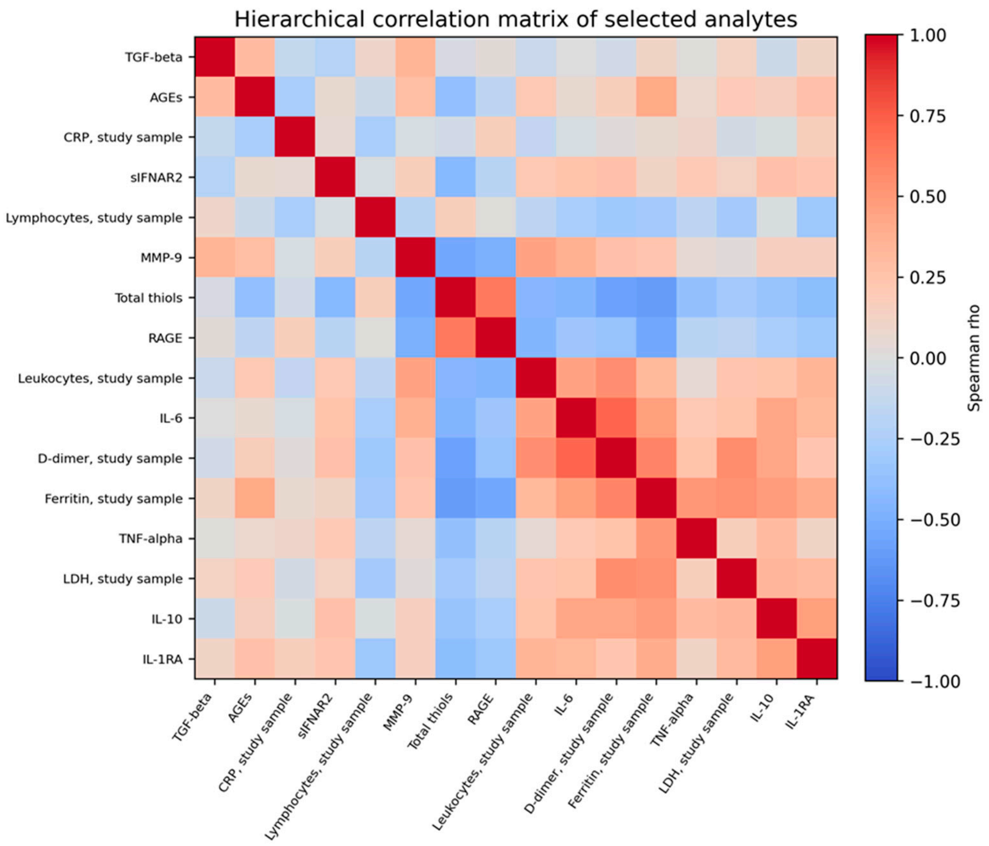

Supplementary Figure S10. Hierarchical Spearman correlation matrix of selected analytes. The matrix illustrates correlation structure among cytokine, thrombo-inflammatory, redox, endothelial/matrix and interferon-axis markers.

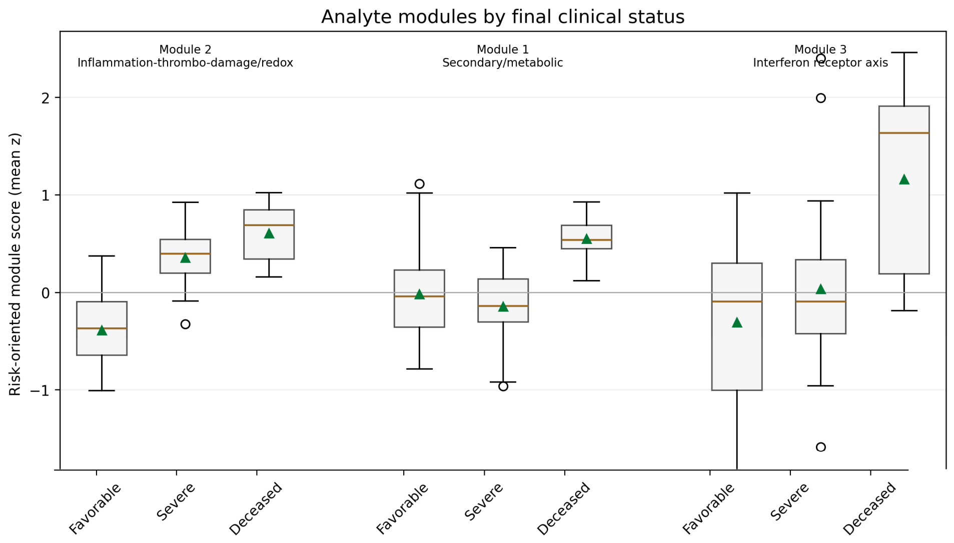

Supplementary Figure S11. Distribution of risk-oriented module scores by final clinical status. Higher scores indicate greater risk-oriented intensity after standardization and direction harmonization.

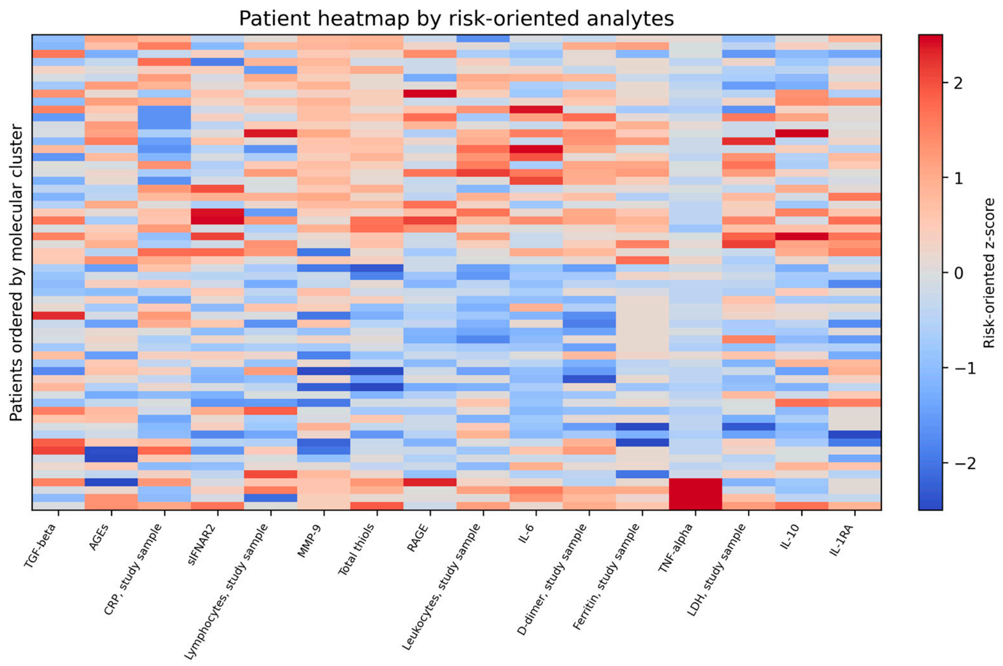

Supplementary Figure S12. Patient-level heatmap by risk-oriented analytes. Rows represent patients ordered by molecular clustering and columns represent standardized analytes. Colour scaling is marker-specific and normalized within each biomarker; colour intensity therefore reflects the relative position of each patient within a given marker only and does not imply comparable effect sizes or a common severity gradient across biomarkers.

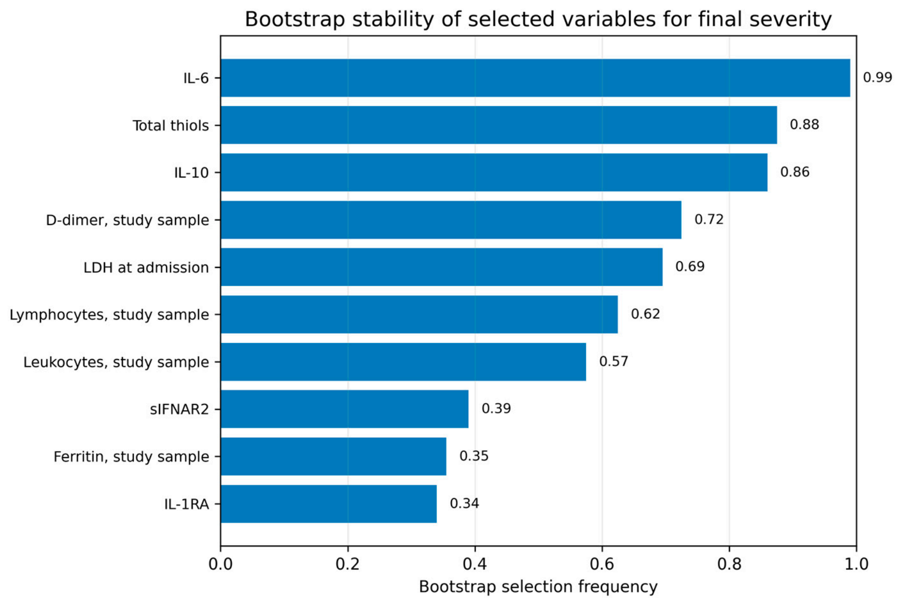

Supplementary Figure S13. Bootstrap stability of LASSO-selected variables for final severity. Selection frequencies are intended to support interpretation of variable stability rather than to define a validated predictor set.

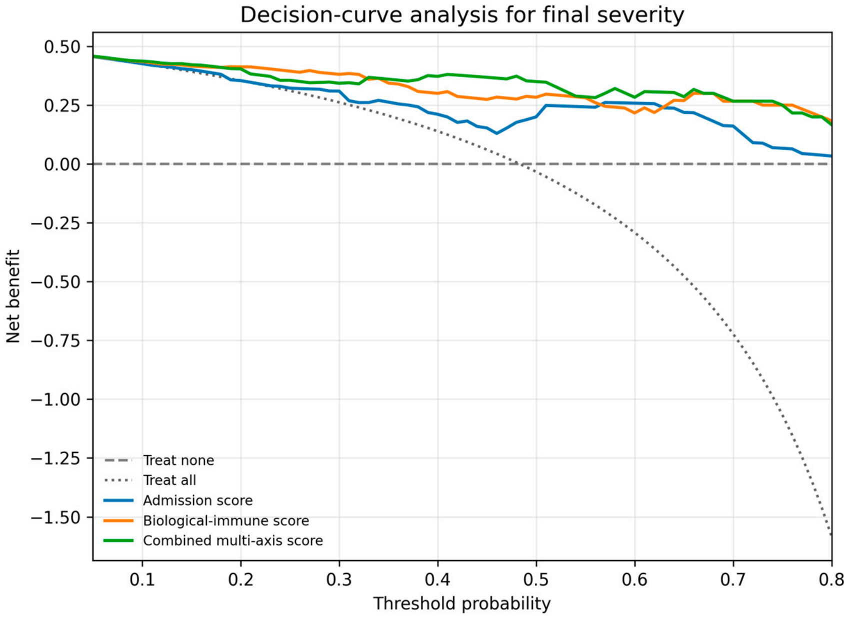

Supplementary Figure S14. Decision-curve analysis for final severity. Net benefit is shown across clinically plausible threshold probabilities for selected exploratory models.

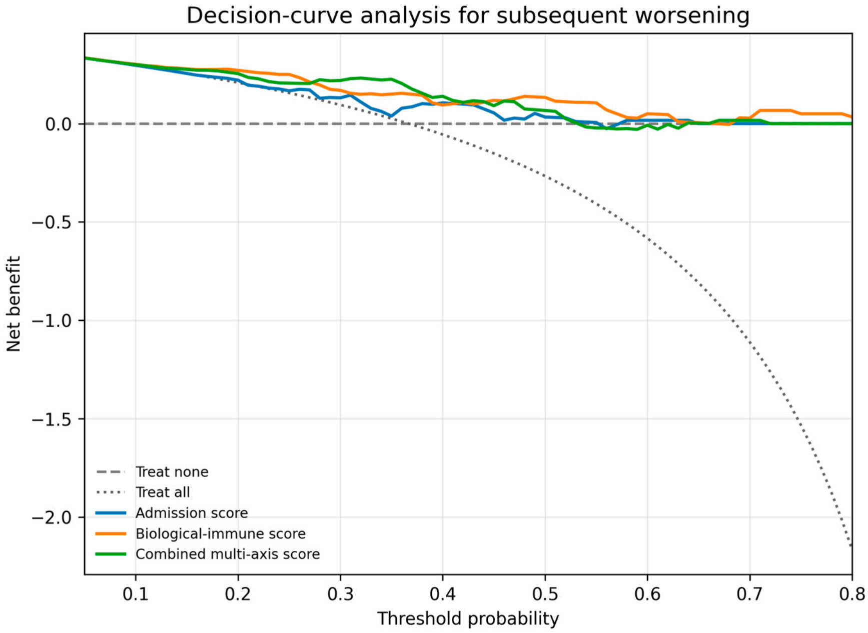

Supplementary Figure S15. Decision-curve analysis for subsequent worsening after the study sample. Net benefit estimates should be interpreted as exploratory and internally derived.
